# Associations Between Daily EMA of Emotional Exhaustion and Monthly Burnout Symptoms During Work Reintegration: Longitudinal Multilevel Study

**DOI:** 10.2196/92458

**Published:** 2026-10-13

**Authors:** Lea Berkemeier, Wim Kamphuis, Hilbrand Oldenhuis, Rudolf Verdaasdonk, Lisette van Gemert-Pijnen

**Affiliations:** 1 Department of Psychology, Health and Technology, Behavioral, Management and Social Sciences (BMS), University of Twente, Drienerlolaan 5, Enschede, Overijssel, 7522 NB, The Netherlands, 31 53 489 9111; 2 Research Group Digital Transformation, Hanze University of Applied Sciences, Groningen, Groningen, The Netherlands; 3 Department of Learning and Workforce Development, TNO, Netherlands Organisation for Applied Scientific Research, Soesterberg, Utrecht, The Netherlands; 4 TechMed Center, University of Twente, Enschede, Overijssel, The Netherlands

**Keywords:** ecological momentary assessment, burnout symptoms, multilevel modeling, longitudinal monitoring, emotional exhaustion, within-person change

## Abstract

**Background:**

Burnout is increasingly conceptualized as comprising both stable, slowly changing features and more dynamic, fluctuating features, with emotional exhaustion widely regarded as one of burnout’s core components. Traditionally, burnout has been assessed using retrospective self-report questionnaires, such as the Burnout Assessment Tool (BAT), administered at sporadic intervals. In contrast, ecological momentary assessment (EMA) enables high-frequency assessment of daily experiences while reducing recall bias. However, it is not well understood how EMA-based measures fluctuate and develop longitudinally within individuals or how they relate to retrospective burnout measures.

**Objective:**

This paper examined the relationship between 30-day aggregates of daily EMA-assessed emotional exhaustion, specifically the within-person mean level and variability (SD), and monthly burnout symptoms measured with the BAT.

**Methods:**

Eighteen participants undergoing work reintegration after stress- or burnout-related sick leave completed daily EMAs of emotional exhaustion (N=3157) and monthly BAT questionnaires over 6 months (N=94). For each monthly BAT assessment, EMA data from the preceding 30 days were aggregated into within-person mean and SD values. Linear mixed-effects models with random intercepts and time effects were fitted separately for each BAT subscale. Additionally, intraclass correlation coefficients (ICCs) were calculated to quantify the proportion of variance attributable to between-person differences.

**Results:**

ICCs indicated that a large proportion of variance in the BAT subscales was attributable to between-person differences, whereas daily EMA emotional exhaustion showed relatively greater within-person variability. Significant time-related changes were observed for the BAT subscales of Cognitive Impairment, Mental Distance, and Emotional Impairment. Within-person increases in the EMA mean of emotional exhaustion were significantly associated with higher scores on BAT Exhaustion (*β*=0.073; *P*=.001) and BAT Cognitive Impairment (*β*=0.041; *P*=.04). EMA-based variability (SD) and between-person mean levels were not significantly associated with any BAT subscale. Conditional *R*^2^ values were high (0.667-0.777), whereas marginal *R*^2^ values were modest (0.056-0.121). Consistent with the ICC findings, these results suggest that most explained variance in BAT scores reflects between-person differences and time-related changes, with EMA-derived predictors accounting for only a limited additional proportion of explained variance.

**Conclusions:**

EMA-based aggregates showed partial correspondence with monthly burnout scores. Within-person increases in mean daily emotional exhaustion were associated with higher subsequent scores on the BAT Exhaustion and Cognitive Impairment subscales, but EMA-based variability was not associated with any BAT subscale. Together with the ICC results, these findings suggest that EMA-based aggregates of emotional exhaustion can reflect short-term fluctuations and longer-term changes in symptom severity of exhaustion and cognitive impairment. Retrospective questionnaires remain useful for global symptom assessment or diagnoses at specific time points but appear less well suited for tracking within-person changes and supporting personalization. These results highlight the importance of aligning the choice of assessment with temporal and clinical goals.

## Introduction

### Background

Burnout has emerged as a major international occupational health concern, with approximately 25% of employees reporting burnout-related symptoms [1,2]. According to Schaufeli et al [3], burnout can be defined as “a work-related state of exhaustion that occurs among employees, which is characterized by extreme tiredness, reduced ability to regulate cognitive and emotional processes, and mental distancing.” Burnout is understood to develop gradually through prolonged exposure to chronic work-related stress, particularly when job demands consistently exceed an individual’s resources to recover and adapt [3]. Rather than resulting from isolated stressful events, burnout is thought to emerge through the cumulative effects of repeated demands combined with insufficient recovery, ultimately leading to poor work performance and feelings of incompetence [3]. This conceptualization is consistent with the Job Demands-Resources model, which proposes that sustained high job demands in combination with insufficient job resources contribute to the development of burnout [4]. Although burnout is recognized as a multidimensional work-related syndrome, emotional exhaustion is widely regarded as the core component of burnout, defined as “feelings of being overextended and depleted of one’s emotional and physical resources” [5]. Since emotional exhaustion is highly responsive to changes in work demands and recovery opportunities, it provides a particularly sensitive indicator of changes in employees’ well-being over time.

The dynamic nature of emotional exhaustion is especially apparent during return-to-work following burnout. Recovery from burnout is increasingly conceptualized as a gradual and dynamic process rather than a discrete transition from illness to health. Clinical models distinguish several overlapping phases of recovery that differ in symptom presentation and rehabilitation goals [6]. During the initial crisis phase, individuals typically experience severe fatigue, emotional distress, cognitive impairments, and markedly reduced functioning, with treatment primarily focusing on recovery from prolonged stress and restoration of basic functioning. In the subsequent recovery phase, individuals gradually resume daily activities and work participation while learning to balance demands and recovery; reestablish healthy routines; and improve sleep, physical activity, and other lifestyle factors [6,7]. This phase often extends over several months and is characterized by considerable within-person fluctuations in exhaustion as individuals adjust to changing demands. Finally, the prevention phase focuses on identifying and addressing the underlying contributors of burnout in order to reduce the risk of relapse [6]. These recovery trajectories suggest that emotional exhaustion is unlikely to decline linearly over time. Since emotional exhaustion is both central to the burnout construct and highly sensitive to daily demands and recovery, its monitoring can provide valuable insights into recovery processes during return-to-work trajectories after burnout, which are known to be dynamic and highly individual [8-11]. Traditionally, burnout has been conceptualized as a relatively stable construct and is typically assessed using retrospective self-report questionnaires administered at relatively sparse time intervals (eg, Xanthopoulou and Meier [12]). However, other research increasingly suggests that well-being and burnout comprise both a relatively stable component that changes slowly over time and a more dynamic component that fluctuates within persons in response to changing circumstances or events [11-14]. With the widespread availability of smartphones, these dynamic components can now be captured in daily life through ecological momentary assessments (EMAs) in a feasible manner. By measuring experiences close in time to their occurrence, EMA reduces recall bias and improves ecological validity [15,16]. EMA is now widely used across mental health research, including in studies of stress, mood, and suicidal ideation, because it captures temporal within-person dynamics that are largely invisible to retrospective methods [17-21].

Importantly, existing within-person research on the dynamic component of burnout has often adopted a differential approach, focusing on momentary covariation between several proximal predictors such as workload or stressors at specific moments in time (see Frick et al [11]). In contrast, a temporal perspective focuses on how burnout-related experiences evolve over time, their typical ebb and flow, their structural patterns across days or weeks, and the extent to which they show stable versus dynamic components. This temporal perspective is essential for developing theories that explain change processes over time and for distinguishing meaningful change from natural fluctuations [11].

A recent experience-sampling study by Frick et al [11] with healthy individuals provided an important first step in this direction by examining momentary burnout-related experiences over 1 week. The results showed that half of the variance in exhaustion was attributable to within-person and across-moment variation, with the other half to between-person differences. This finding supports a shift away from a purely trait-like conceptualization of burnout and demonstrates that burnout-related experiences may fluctuate substantially within days and individuals. Although Frick et al [11] and earlier diary studies (eg, Sonnenschein et al [22]) demonstrated that exhaustion fluctuates substantially within individuals, these studies were limited to short observation windows, typically 1 or 2 weeks long. As a result, it remains unclear how such momentary fluctuations accumulate, stabilize, or change over longer periods, such as during recovery from burnout, and how they relate to traditional measures that aim to capture more stable aspects of burnout. Understanding the temporal relationship between EMA-based and retrospective self-report measures of burnout can help practitioners interpret the results from each measurement approach. Moreover, it can inform future study designs, including decisions regarding assessment frequency and time scale.

### EMA Features and Affect Dynamics

There is growing interest in how emotions unfold and fluctuate over time, so-called affect dynamics, and how these processes relate to psychopathology [23] and well-being [20,24]. The most widely used summary features of EMA time series are the mean level and variability, typically operationalized as the intraindividual SD. The mean reflects a person’s typical level of affect or symptoms and is often interpreted as a trait-like indicator [25]. Variability reflects the extent to which experiences fluctuate over time and is often interpreted as an index of emotional instability or lability [20,26,27]. Both features have demonstrated clinical relevance. Average affect levels have been linked to depression, anxiety, posttraumatic stress disorder, and borderline personality disorder, while higher affective variability has been associated with lower well-being and greater psychopathology (see Pirla et al [25] for a review).

In contrast, more complex temporal indicators such as inertia or autocorrelation have shown mixed and often small incremental predictive value. Inertia refers to the tendency of an emotional state to persist over time, such that a person’s emotional state at one moment influences their emotional state at the next [20,28]. Statistically, inertia is commonly operationalized as the autocorrelation of a time series, with higher autocorrelation indicating slower recovery from emotional experiences or greater persistence of emotional states [20,28]. Although some studies report associations of these indices with depressive symptoms (eg, [29,30]), a large and influential study by [20] demonstrated that such complex dynamics add little predictive value beyond mean levels for well-being. Methodological reviews further caution that many dynamic indices suffer from substantial reliability and design-dependence problems [25,31,32]. For these reasons, recent recommendations emphasize prioritizing robust and interpretable EMA features, particularly mean levels and variability. Accordingly, the present study focuses on these 2 features as the most defensible candidates for linking daily EMA measurements to monthly retrospective measurements.

### Gaps and Aims

Despite the important insights, existing EMA studies on burnout or occupational reintegration focused on short time windows ranging from a few days to several weeks [11,22]. While such designs are well-suited for identifying micro-temporal patterns within days or weeks, they are limited in their ability to capture slower, nonlinear change processes that unfold over months, such as recovery, relapse, plateaus, or longer-term adaptation. A small number of studies outside the occupational reintegration context have begun to extend EMA designs to longer periods (eg, Webb et al [33], Morgan et al [34]), but even these typically span only a few weeks to 3 months. Moreover, Pirla et al [25] showed that for nontemporally dependent features such as mean and variability, temporally dispersed sampling over longer periods can yield more accurate estimates than dense sampling over short periods. Together, these considerations suggest that longer-term EMA designs may be particularly well suited to studying how aggregated momentary experiences relate to broader retrospective summaries.

The present study leverages a naturalistic occupational rehabilitation context in which substantial within-person change is expected. Emotional exhaustion and burnout-related symptoms are known to fluctuate over time, particularly during return-to-work trajectories; yet, to the best of the authors’ knowledge, no intensive longitudinal study has examined affect dynamics in this context over several months. Furthermore, the study is explicitly grounded in the idiographic, within-person perspective emphasized in modern ambulatory assessment methodology [35,36]. Instead of asking how people differ from each other, the focus is on how each person’s daily experiences relate to their monthly symptom reports over time. To link these momentary experiences to retrospective assessments in a robust and interpretable way, the present study follows recommendations from the affect dynamics literature and focuses on 2 well-established EMA summary features, namely the mean level and variability (SD).

The current study addresses the following research questions:

1. To what extent are within-person variations in the mean level of daily emotional exhaustion, measured by EMA, associated with retrospective burnout scores?
2. To what extent is the variability (SD) of daily emotional exhaustion, measured by EMA, associated with retrospective burnout scores beyond the mean level?

## Methods

### Study Design and Setting

The observational study examined the correspondence between daily EMAs of emotional exhaustion and monthly burnout symptoms over a 6-month period of work reintegration. In this context, correspondence refers to the extent to which aggregated daily EMA features (eg, mean level, variability) align with monthly questionnaire scores within the same individual. To address the research questions, daily EMA data were aggregated to monthly questionnaire assessments. The primary analytic focus was within-person to examine whether months in which an individual reported a higher-than-usual average of emotional exhaustion were also months with higher burnout scores, and whether within-person variability (SD) in daily emotional exhaustion was associated with retrospective burnout scores beyond the mean level.

### Ethical Considerations

The study was reviewed and approved by the Behavioral, Management, and Social Sciences (BMS) ethical committee of the University of Twente (request number 241104). This study was part of a larger project on monitoring return-to-work trajectories using passive sensing. However, the present analyses focus exclusively on the relationship between daily EMA of emotional exhaustion and monthly burnout questionnaire scores. All participants provided written informed consent prior to participation. Consent procedures covered the study aims, daily EMA and monthly questionnaire assessments, data management procedures, and the broader multimodal sensing project. Data were collected in a pseudonymized form during the study and anonymized after data collection was completed. Personal identifiers were stored separately from research data and accessible only to the main researcher during the data collection but were deleted after study completion. These procedures ensured that research data could not be traced back to participants once the study concluded. In terms of compensation, participants who filled in at least 85% valid EMA surveys were allowed to keep the Oura Ring that they used during the data collection.

### Participants and Procedure

Participants were recruited through occupational physicians at ArboNed B.V., the largest occupational health service in the Netherlands. These physicians oversee employees on long-term sick leave and initiate return-to-work trajectories, providing a clinically relevant setting for studying fluctuations in emotional exhaustion during recovery. Eligible individuals were employees who had been formally assessed by an occupational physician within the first 6 weeks of work absence and diagnosed with stress- or burnout-related symptoms. At the time of recruitment, all participants had recently begun a clinician-guided return-to-work trajectory, a period during which meaningful variation in exhaustion across weeks and months was expected. Under Dutch occupational health legislation (“Wet verbetering poortwatcher”), employees on long-term sick leave are legally required to participate in a structured reintegration process coordinated by an occupational physician. These counseling sessions were not part of the study protocol and were not influenced by the research team. Because the study was observational and conducted in a naturalistic context, counseling sessions formed part of the environment in which emotional exhaustion was measured over time. Their potential influence on outcomes is therefore inherent to the ecological nature of the study rather than a controlled or experimental component.

Nineteen employees met initial eligibility criteria and were enrolled across 2 cohorts: 10 participants started in November 2024 (followed until June 2025), and 9 participants started in January 2025 (followed until August 2025). To be included in the analyses, participants were required to provide at least 85% of valid daily EMAs (approximately 156 points, corresponding to 5.1 months) and at least 5 out of 6 monthly questionnaire assessments. In addition to the 19 individuals, an additional 20 individuals indicated interest in participating in the study. Of these 20 individuals, 6 did not respond to contact initiation about an intake interview, 11 declined the intake interview after receiving detailed information about the study and 3 showed interest but were already further in their return-to-work trajectory and thus did not meet the inclusion criteria. One enrolled participant (ID 12) discontinued participation after 3 weeks due to discomfort of wearing a smart ring (see Berkemeier et al [37]), resulting in a final sample of 18 participants.

Individuals learned about the study through recruitment flyers distributed by occupational physicians. Interested employees received detailed written information about the study aims, procedures and data management. They subsequently completed an online or in-person intake interview with the main researcher. During this intake, participants provided written informed consent, which explicitly covered all data collection and processing procedures. Inclusion criteria were as follows: (1) being on long-term sick leave (>6 wk) due to stress- or burnout-related symptoms, diagnosed by an occupational physician within the first 6 weeks of sick leave; (2) being aged 18 to 67 years; (3) being at the start of a clinician-guided return-to-work trajectory; (4) willingness and ability to complete daily EMA and monthly assessments for 6 months; and (5) sufficient Dutch language proficiency to complete all assessments. Although the broader project required participants to wear an Oura ring and allow passive smartphone data collection, these sensing data are not used in the present analyses. The broader project is described in detail in a previously published article [38]. The current study uses the same participant cohort but focuses exclusively on daily EMA of emotional exhaustion and monthly burnout questionnaires. Exclusion criteria were determined by the occupational clinician and included severe medical or psychiatric conditions that could interfere with, distort, or confound physiological measurements collected in the broader multimodal sensing project (eg, sleep and heart-rate metrics from the Oura ring). These conditions included heart rhythm disorders, usage of antiarrhythmic medication, kidney and/or renal dysfunction, diabetes mellitus, previously isolated diagnosis of posttraumatic stress disorder, depression, or anxiety disorder, or substance dependence.

Participants used the Avicenna smartphone app [38] to complete daily EMAs of emotional exhaustion and monthly burnout questionnaires. Although the broader project involved passive sensing data from the smartphone to monitor return-to-work trajectories (see Berkemeier et al [37]), the present analyses focus solely on the relationship between daily emotional exhaustion and monthly burnout symptoms.

### Measures

#### Daily Emotional Exhaustion (EMA)

Participants completed 1 daily time-contingent EMA item assessing emotional exhaustion via the Avicenna app: “At this moment, how emotionally exhausted do you feel” (see Carson et al [39]). Responses were provided on a visual analog scale from 0 (not at all) to 100 (extremely). To ensure consistent end-of-day reporting, participants selected a fixed daily notification time between 5:00 PM and 11:00 PM. This restricted evening window was chosen to understand how emotionally exhausted a person felt at the end of the day after all their activities during the day. Participants received 1 notification per day at their chosen time. To accommodate variations in daily routines, the EMA item remained available for 12 hours after the initial prompt. If the question had not been answered within 2 hours, the Avicenna app sent another reminder notification. This design balanced protocol consistency with participant flexibility, ensuring that assessments occurred at a suitable moment while still anchoring each rating to the same general time frame across days.

#### Monthly Burnout Assessment Tool

Once per month, participants completed the validated short general version of the Burnout Assessment Tool (BAT) [3,40] via the Avicenna app. The general version of the BAT was selected because participants were enrolled in a return-to-work trajectory, meaning that not all participants had fully resumed work at the time of assessment. The BAT contains 12 items to assess the core dimensions of burnout and comprises 4 subscales: Exhaustion, Mental Distance, Cognitive Impairment, and Emotional Impairment. The Exhaustion subscale assesses severe physical and mental fatigue (eg, “I feel mentally exhausted”). The Mental Distance subscale assesses psychological detachment and reduced interest or enthusiasm (eg, “I struggle to find any enthusiasm for my work”). The Cognitive Impairment subscale measures difficulties in attention, concentration, and memory (eg, “I have trouble staying focused”). Finally, the Emotional Impairment subscale assesses difficulties regulating emotions, such as becoming irritated or overreacting more easily than usual (eg, “I overreact unintentionally”). Participants evaluated their burnout symptoms on a 5-point Likert scale ranging from 1 (“never”) to 5 (“always”). In the present study, the 4 BAT subscales were used as separate outcome measures to examine how different aspects of burnout relate to EMA-derived features of daily emotional exhaustion. All four subscales showed good internal consistency (α>.70; [41]; Table 1).

**Table 1.** Internal consistency of Burnout Assessment Tool (BAT) subscales during work reintegration.

Questionnaire, scale, and subscale	Number of items	Cronbach α of other studies	Cronbach α of current study
Burnout Assessment Tool			
Five-point Likert from never (1) to always (5)			
Exhaustion	3	0.87 [3]	0.85
Cognitive Impairment	3	0.92 [3]	0.89
Emotional Impairment	3	0.90 [3]	0.92
Mental Distance	3	0.90 [3]	0.82

### Data Preparation

To align the EMA aggregation period with the monthly assessment interval while maximizing data coverage and estimate stability, a 30-day look-back window was selected for the main analyses. Because BAT scores were collected once per month, EMA features needed to be represented at the same temporal resolution to be able to relate predictors to outcomes. Daily EMA observations cannot be used directly as predictors of monthly BAT scores without violating this alignment or inflating the number of predictor observations relative to outcome observations. Instead, all daily observations within each 30-day window contributed to the computation of the monthly mean and monthly SD of emotional exhaustion.

Similarly, research by [25] also highlighted that 30 observations per individual, across windows ranging from 3 to over 30, yield the most reliable estimates of affect dynamics (average and SD) and the lowest root mean squared error. For each person × month window, two EMA-derived features were computed: (1) the mean level of emotional exhaustion and (2) the SD of emotional exhaustion. The mean was further decomposed into the person-specific average across all windows (between-person) and the deviation of each window from the person’s own mean (within-person). This decomposition (see Myin-Germeys et al [42]) ensures that within-person coefficients reflect month-to-month changes within individuals, whereas between-person coefficients capture stable individual differences (to be interpreted cautiously given the small sample size). Variability (SD) was treated as a time-varying window-based feature and was not further decomposed.

Since descriptive plots suggested potentially nonlinear symptom trajectories across the reintegration months, several time specifications were compared for each BAT subscale: a linear time model, a quadratic time model, and a flexible spline time model. Model comparisons using likelihood ratio tests (machine learning estimation) indicated that only BAT Exhaustion showed a significantly better fit for the spline specification, whereas Cognitive Impairment, Emotional Impairment, and Mental Distance were adequately captured by linear time trends. Accordingly, the final models included a spline-based time term for Exhaustion and linear time terms for the other subscales. Primary analyses were conducted using complete-case multilevel models. Because some monthly questionnaire outcomes were missing, a sensitivity analysis using multilevel multiple imputation (20 imputations) was performed for the outcome variables, with participants specified as the clustering level. Imputation models included time and all EMA-derived predictors. The results from the imputed analyses were highly similar to the complete-case analyses, and all substantive conclusions were unchanged. Therefore, only the complete-case models are reported.

### Statistical Analysis

All analyses were conducted in RStudio (version 2024.12.1 Build 563) using R (version 4.4.1), and the full R Markdown can be found in Multimedia Appendix 1. First, descriptive statistics were computed for all EMA-derived predictors and BAT subscales, including means, SDs, and ranges. To do so, statistics were first computed within each person over their full time series, and those person-level statistics were then summarized across persons, as suggested by Siepe et al [43]. To obtain an initial overview of the associations between EMA features and burnout subscales, Pearson correlations between the EMA-derived predictors (within-person mean and SD) and each BAT subscale were computed. Statistical significance was evaluated using a 2-sided α level of .05. To quantify the proportion of variance attributable to stable between-person differences versus within-person fluctuations, intercept-only multilevel models were fitted to daily EMA data on emotional exhaustion and to each monthly BAT subscale. For each outcome, the intraclass correlation coefficient (ICC) was computed as the ratio of between-person variance in total variance, with values closer to 1 indicating predominantly between-person (stable component) variation, whereas lower values indicate greater within-person (dynamic component) fluctuation over time.

Second, to answer the research questions, linear mixed-effects models were estimated with fixed effects for time and the EMA-derived predictors (within-person mean, between-person mean, and SD) and a random intercept for participant. Model assumptions were visually and statistically inspected, with no evidence of problematic outliers or multicollinearity. The within-person mean predictor tested whether months in which a person reports higher-than-usual daily emotional exhaustion are also months with higher burnout scores (research question 1). The SD predictor tests whether month-to-month differences in daily variability in emotional exhaustion explain additional variance beyond mean levels (research question 2). The between-person mean was included to control for stable individual differences in overall exhaustion level. Models were fitted using maximum likelihood estimation. For each model, the fixed-effects estimates, *P* values, and 95% CIs were reported. Model explanatory power was summarized using Nakagawa’s marginal *R*^2^ (variance explained by fixed effects, thus emotional exhaustion predictors and time) and conditional *R*^2^ (variance explained by fixed and random effects combined).

## Results

### Descriptives

Participant characteristics are presented in Table 2. Across the full study period, 3595 EMA surveys were issued. Of these, 3157 (87.82%) observations were valid ratings of emotional exhaustion, and 438 (12.18%) were missing and excluded. The number of valid observations per participant ranged from 136 (72.60%, participant 16) to 191 (95.50% participant 3). Across all daily EMAs, emotional exhaustion showed a moderate average level (mean 42.9) with substantial variability (SD 15.5).

**Table 2.** Characteristics of 18 participants undergoing work reintegration following stress- or burnout-related sick leave.

Characteristics	Values
Sex, n (%)	
Male	8 (44)
Female	10 (56)
Age (y), mean (SD)	39.6 (12.1)
Profession, n (%)	
Manager	4 (22.2)
Consultant	3 (16.7)
Human resources	2 (11.1)
Other	8 (44.4)
Not available	1 (5.6)
Educational level, n (%)	
Primary school	1 (5.6)
Secondary school	2 (11.1)
Technical secondary vocational education	1 (5.6)
University of applied sciences bachelor	8 (44.4)
University of applied sciences master	1 (5.6)
University bachelor	1 (5.6)
University master	3 (16.7)
University diploma	1 (5.6)
Counseling sessions with occupational physician, n (%)	
One session	4 (22.2)
Two sessions	9 (50)
Three sessions	3 (16.7)
Four sessions	2 (11.1)

As shown in Figure 1, daily emotional exhaustion levels and trajectories varied substantially both within and between individuals across the 6-month period. For example, when zooming in on 1 month of observations, participant 18 and participant 7 have the same average of emotional exhaustion in that month (mean 39.8); however, they differ widely in the variability of their observations, with an SD of 12.7 for participant 18 and an SD of 26.3 for participant 7 (Figure 2). The results of the variance decomposition analysis showed an ICC of 0.57 for daily EMA emotional exhaustion, indicating that approximately 57% of the variance in emotional exhaustion reflected stable between-person differences, whereas 43% reflected within-person day-to-day fluctuations.

**Figure 1.**
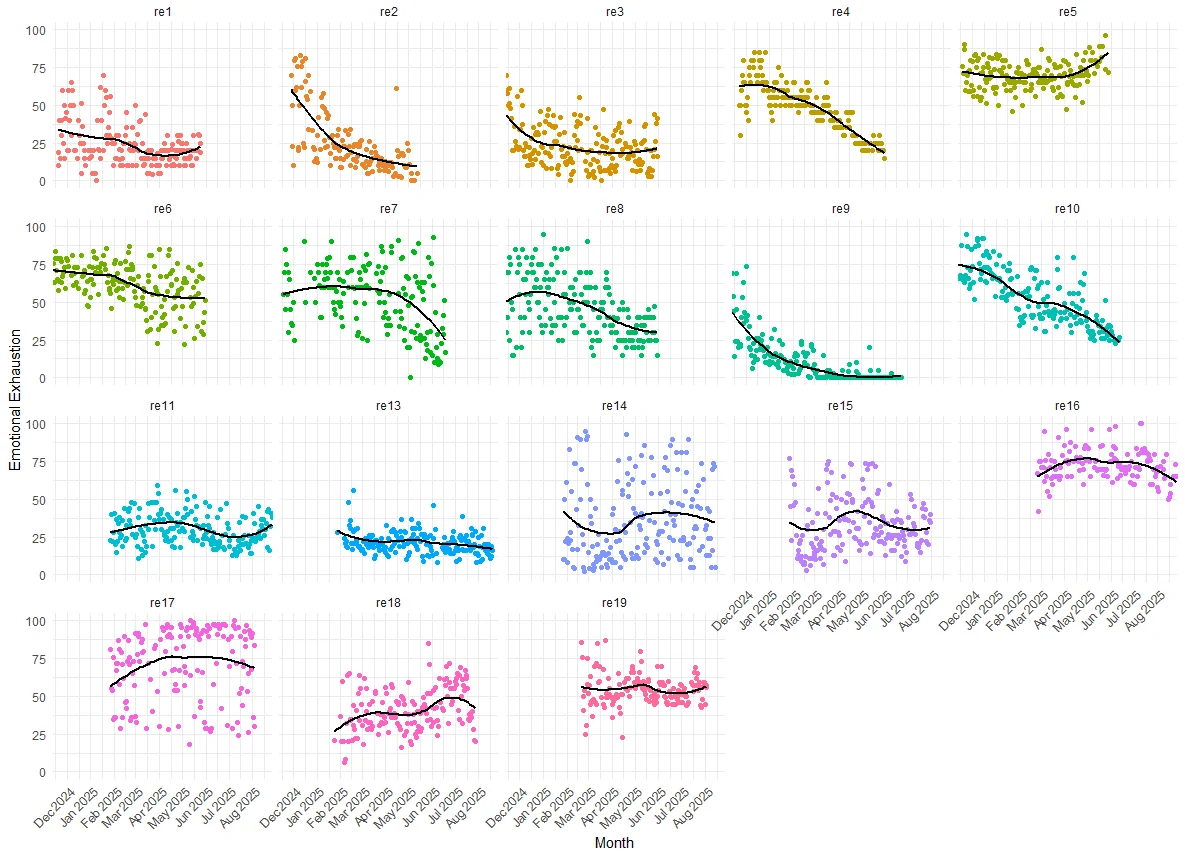
Daily emotional exhaustion trajectories during a 6-month work reintegration period. Individual trajectories of daily emotional exhaustion were assessed using ecological momentary assessment (EMA) among 18 participants on long-term sick leave due to stress- or burnout-related symptoms undergoing clinician-guided work reintegration in the Netherlands. Participant 12 was excluded from the analyses.

**Figure 2.**
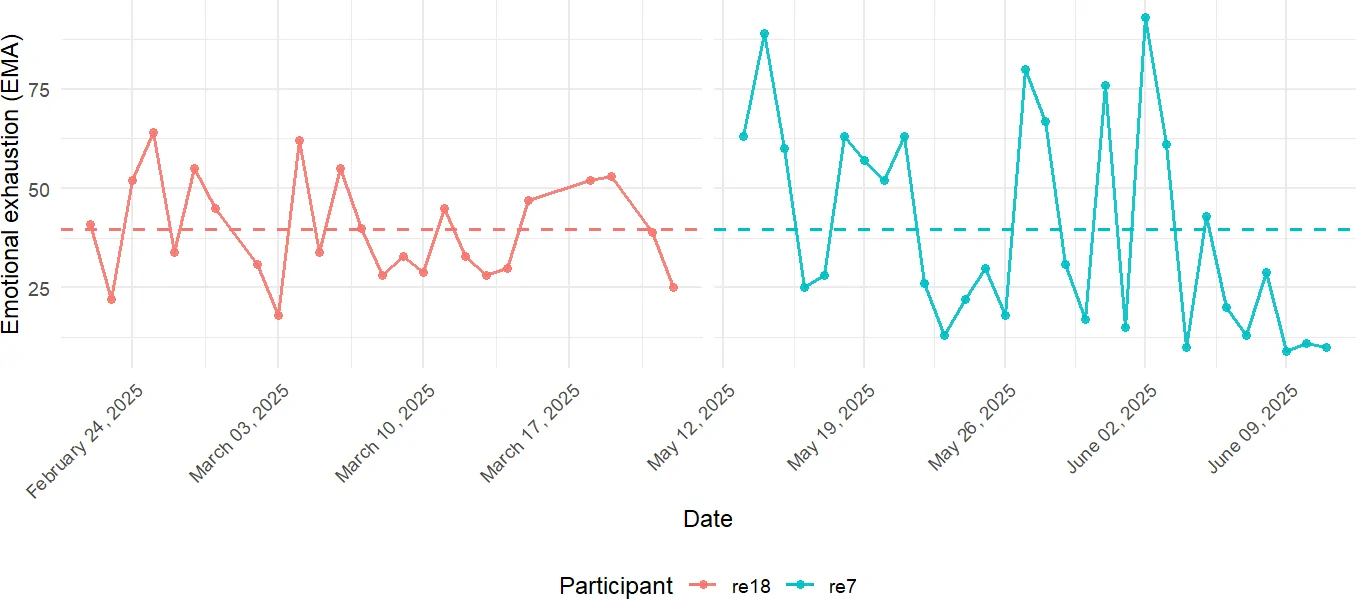
Comparison of monthly emotional exhaustion patterns between 2 participants during work reintegration. Solid line indicates daily observations; dashed line indicates mean level within the window. Monthly observations of emotional exhaustion are shown for 2 participants to illustrate individual differences in symptom levels and changes over the 6-month work reintegration period. EMA: ecological momentary assessment.

For the monthly retrospective questionnaires, 94 valid observations out of the 123 observations (76.4%) were available. The BAT subscales showed mean scores of 8.95 (SD 1.33) for Exhaustion, 7.90 (SD 1.27) for Cognitive Impairment, 7.28 (SD 1.43) for Emotional Impairment, and 7.81 (SD 1.66) for Mental Distance. Similar to the patterns of emotional exhaustion, the monthly BAT subscale scores (Figure 3) also showed considerable interindividual differences and intraindividual change over time. For the monthly BAT subscales, variance decomposition analyses showed that the ICCs ranged from 0.56 (Mental Distance), 0.68 (Emotional Impairment), 0.71 (Exhaustion) to 0.74 (Cognitive Impairment). This indicates that approximately 56% to 74% of the variance in BAT subscale scores reflected stable differences between individuals, with the remaining 26% to 44% reflecting within-person fluctuations across measurements.

**Figure 3.**
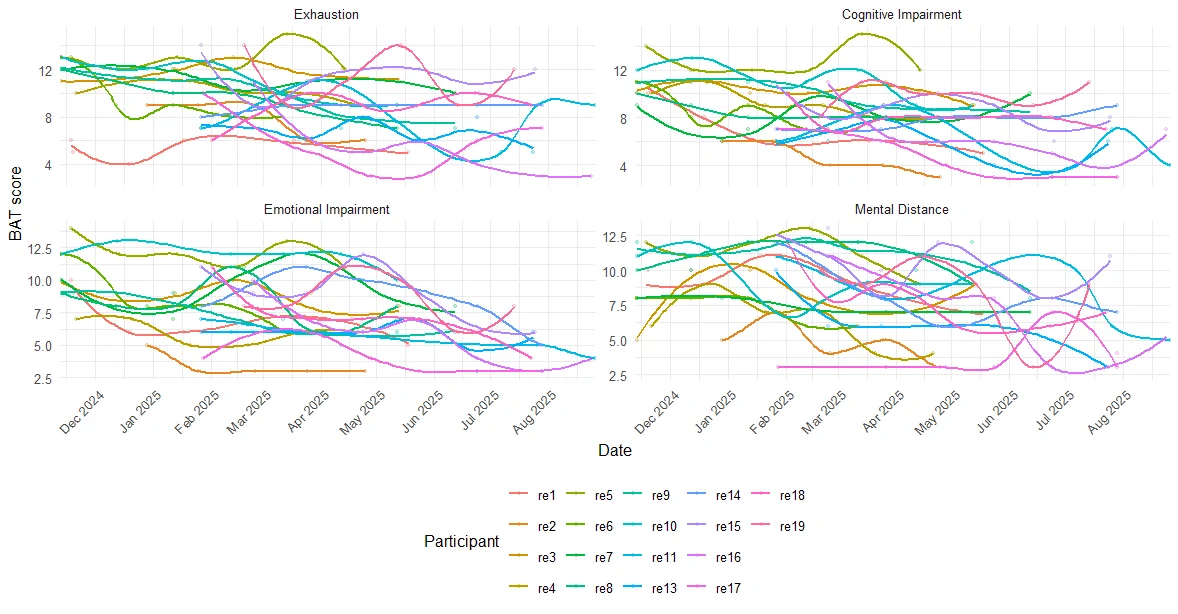
Burnout Assessment Tool (BAT) subscale scores across the 6-month work reintegration period. Monthly scores for the 4 Burnout Assessment Tool (BAT) subscales among 18 participants on long-term sick leave due to stress- and burnout-related symptoms undergoing clinician-guided work reintegration in the Netherlands. Participant 12 was excluded from the analyses.

Bivariate correlation analyses (Figure 4) indicated that the within-person mean level of daily emotional exhaustion was significantly and positively associated with BAT Exhaustion (*r*=0.22; *P*=.04) and BAT Cognitive Impairment (*r*=0.21; *P*=.04), indicating that months characterized by higher-than-usual daily exhaustion were also months in which participants reported higher levels of these burnout symptoms. For BAT Emotional Impairment (*r*=0.11; *P*=.29) and BAT Mental Distance (*r*=0.16; *P*=.13), the within-person mean level of daily emotional exhaustion was also positively associated, but nonsignificantly. Associations between the within-person variability (SD) emotional exhaustion and the BAT subscales were also nonsignificant. For BAT Cognitive Impairment, the correlation was nonsignificant (*r*=−0.12; *P*=.23) and for BAT Emotional Impairment, the association was also nonsignificant (*r*=0.07; *P*=.47). Correlations with the SD of emotional exhaustion for BAT Exhaustion (*r*=0.003; *P*=.98) and BAT Mental Distance (*r*=−0.01; *P*=.89) were close or equal to 0 and nonsignificant.

**Figure 4.**
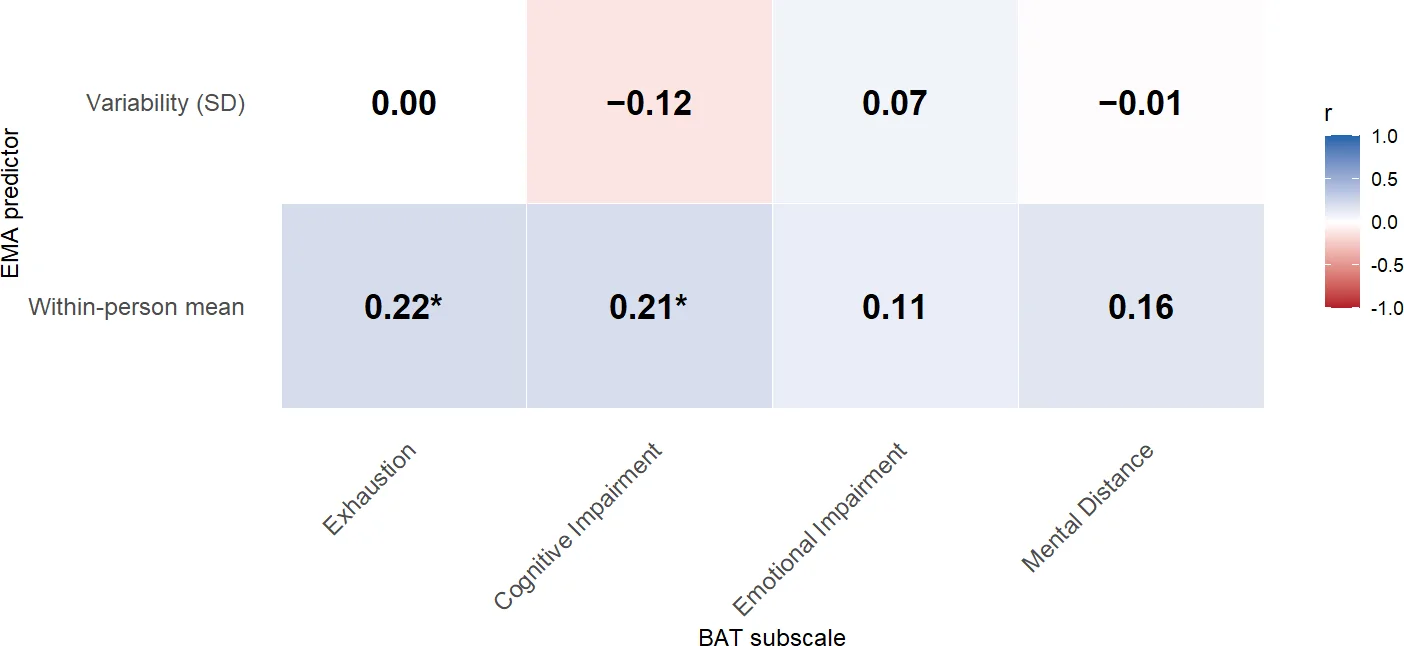
Correlations between ecological momentary assessment (EMA)–derived emotional exhaustion measures and monthly burnout symptoms during work reintegration. Correlations are shown for the examined predictor-outcome combinations between daily EMA measures of emotional exhaustion and monthly Burnout Assessment Tool (BAT) subscale scores over the 6-month study period. *P<.05.

### Linear Mixed Effects Models

#### BAT Exhaustion

For BAT Exhaustion, the spline-based time terms were not statistically significant (*β*=−0.92, 95% CI −2.19 to −0.35, *t*_77.49_=−1.44; *P*=.16), indicating that monthly exhaustion scores did not show a clear systematic time trend over the reintegration period in this sample. The within-person mean level of daily emotional exhaustion was a significant positive predictor of monthly exhaustion scores (*β*=0.07, 95% CI 0.03‐0.12, *t*_78.13_=3.45; *P*<.001). This indicates that during months in which individuals experienced higher-than-usual daily exhaustion, they also reported higher levels of burnout-related exhaustion. Neither the between-person mean level of daily emotional exhaustion (*β*=−0.01, 95% CI −0.07 to −0.05, *t*_18.06_=−0.39; *P*=.70) nor within-person variability (SD; *β*=0.02, 95% CI −0.06 to −0.10, *t*_93.79_=0.44; *P*=.66) showed a significant association with BAT Exhaustion. The fixed effects explained 7.5% of the variance in exhaustion (marginal *R*^2^=0.075), whereas the full model, including random effects, explained 77.7% of the variance (conditional *R*^2^=0.777).

#### BAT Cognitive Impairment

For cognitive impairment, a significant negative linear time effect was observed (*β*=−0.007, 95% CI −0.013 to −0.001, *t*_79.40_=−2.40; *P*=.02), indicating gradual symptom improvement over time. The within-person mean level of daily emotional exhaustion was also a significant positive predictor (*β*=0.041, 95% CI 0.0014‐0.08, *t*_77.90_=2.052, *P*=.04), indicating that months with higher-than-usual exhaustion were associated with more cognitive impairment complaints. Within-person variability (*β*=−0.015, 95% CI −0.09 to -0.06, *t*_92.87_=−0.395; *P*=.69) and between-person mean level (*β*=0.005, 95% CI −0.054 to 0.06, *t*_17.77_=0.168; *P*=.87) were not significantly associated with cognitive impairment. The fixed effects accounted for 5.6% of the variance (marginal *R*^2^=0.056), and the full model explained 77.5% of the variance (conditional *R*^2^=0.775).

#### BAT Emotional Impairment

For emotional impairment, a significant negative linear time trend was observed (*β*=−0.0158, 95% CI −0.02 to −0.009, *t*_79.96_=−4.94; *P*<.001), indicating symptom improvement over time. None of the EMA-derived predictors, within-person mean level (*β*=−0.009, 95% CI −0.08 to −0.03, *t*_78.33_=−0.39; *P*=.70), within-person variability (*β*=−0.02, 95% CI −0.10 to 0.05, *t*_93.62_=−0.62; *P*=.54) nor between-person mean level (*β*=0.02, 95% CI −0.03 to −0.084, *t*_17.98_=0.84; *P*=.41) were significantly related to emotional impairment. The fixed effects explained 12.1% of the variance (marginal *R*^2^=0.121), and the full model explained 76.5% of the variance (conditional *R*^2^=0.766).

#### BAT Mental Distance

Similarly, mental distance showed a significant negative linear time effect (*β*=−0.015, 95% CI −0.02 to −0.008, *t*_81.46_=−4.09; *P*<.001), indicating a decrease in symptoms over time. None of the EMA-derived predictors, within-person mean level (*β*=0.003, 95% CI −0.05 to 0.05, *t*_79.32_=0.116; *P*=.91), within-person variability (*β*=0.016, 95% CI −0.07 to 0.10, *t*_92.49_=0.35; *P*=.73) nor between-person mean level (*β*=−0.02, 95% CI −0.08 to 0.03, *t*_17.94_=−0.89; *P*=.38) was significantly associated with mental distance. The fixed effects explained 12.1% of the variance (marginal *R*^2^=0.121), while the full model explained 66.7% of the variance (conditional *R*^2^=0.667). A summary of the fixed effects across all models is presented in Figure 5.

**Figure 5.**
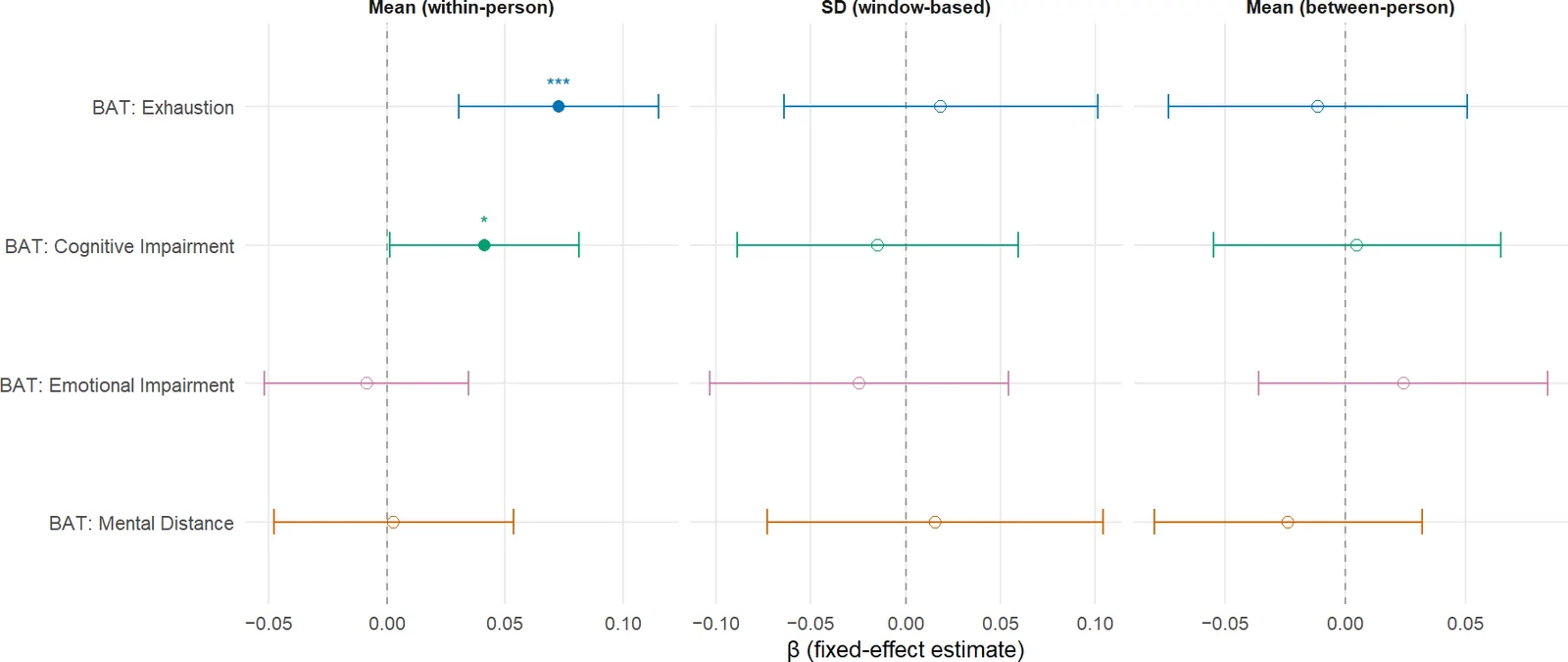
Fixed effects estimates from linear mixed-effects models of monthly burnout symptoms during work reintegration. Window-based SD was calculated over the 30-day period preceding each monthly retrospective assessment. Fixed-effects estimates are shown for models examining associations between within-person mean levels and variability of daily ecological momentary assessment (EMA)–assessed emotional exhaustion and monthly Burnout Assessment Tool (BAT) subscale scores over the 6-month study period. *P<.05, ***P<.001.

## Discussion

### Overview

This study examined how daily emotional exhaustion measured using EMA relates to monthly burnout questionnaire scores during a 6-month work reintegration period. From a within-person perspective, two research questions were addressed: (1) whether within-person changes in the mean level of daily EMA of emotional exhaustion are associated with changes in monthly burnout scores and (2) whether within-person variability in daily EMA emotional exhaustion explains additional variance beyond the mean. Overall, the findings showed that mean levels of daily emotional exhaustion, but not their day-to-day variability, were associated with monthly burnout symptoms, specifically exhaustion and cognitive impairment.

### Main Study Findings

Starting with the first research question, the results showed that higher within-person mean levels of daily emotional exhaustion were linked to higher subsequent monthly BAT Exhaustion and Cognitive Impairment subscale scores. These findings indicate that months characterized by elevated average daily emotional exhaustion corresponded to higher levels of monthly reported exhaustion and cognitive complaints. This pattern aligns with earlier work showing that the mean level of momentary experience or affect is the most robust bridge between EMA and retrospective reports (eg, [20]). From a measurement perspective, this result suggests that aggregated EMA data can capture sustained, clinically meaningful changes in symptom severity over longer intervals, while retaining the advantages of reduced recall bias and greater ecological validity [15,16]. In other words, the result that EMA-derived mean emotional exhaustion tracks monthly exhaustion and cognitive impairment severity suggests that high-frequency, in-the-moment assessments can provide both fine-grained temporal information and longer-term indicators of symptom burden. This is particularly relevant in applied contexts such as occupational reintegration, where continuous monitoring may enable earlier detection of deterioration or stagnation than sparse retrospective assessments.

Turning to the second research question, within-person changes in variability (SD) of daily emotional exhaustion did not explain additional variance in any of the BAT subscales beyond the mean. This finding is in line with a growing literature showing that variability and other more complex dynamic indices often add little incremental predictive value over mean levels [20,25,31,32]. Conceptually, it likely reflects a mismatch between the construct being summarized and the construct being predicted. The BAT measures symptom severity rather than emotional instability or lability, which likely explains why mean levels, but not variability, were associated with monthly exhaustion and cognitive impairment. (eg, Mengelkoch et al [44]). These results do not imply that variability is unimportant. Rather, they suggest that day-to-day variability in emotional exhaustion may capture a different dimension of symptom dynamics that is not well represented in the current instruments used for measuring burnout. In this sense, the present results do not argue against the relevance of variability as a construct of retrospective instability measures.

Furthermore, the variance decomposition results provided crucial context for interpreting these findings. Monthly BAT subscales showed high ICCs (~0.56-0.74), indicating that most of their variance reflects stable between-person differences. In contrast, daily EMA emotional exhaustion showed a substantially lower ICC (~.57) for 3 out of 4 subscales, reflecting greater within-person fluctuation. This difference explains why the multilevel models yielded high conditional *R*^2^ values (driven largely by stable individual differences) but relatively low marginal *R*^2^ values for the EMA-derived predictors and time trends.

In addition to these associations, time trends also contributed meaningfully. Cognitive Impairment, Emotional Impairment, and Mental Distance showed gradual improvement over the course of the reintegration period. In contrast, exhaustion did not exhibit a uniform time trend, suggesting either greater heterogeneity in recovery trajectories or a slower and less uniform recovery process. Overall, the BAT subscales appear to capture slower, macro-temporal changes, while EMA-based emotional exhaustion reflects both gradual shifts and faster, person-specific fluctuations, at least for exhaustion and cognitive impairment severity.

Taken together, the present findings illustrate that the frequency and type of assessments should be aligned with the temporal dynamics of the construct of interest [45,46]. In the present data, monthly burnout questionnaires were primarily dominated by stable differences between individuals, whereas daily EMAs of emotional exhaustion showed more fluctuation within individuals across days. This dissociation illustrates why research focused on within-person mechanisms and person-specific changes over time should not rely solely on sparse retrospective assessments but instead requires intensive longitudinal methods [35,36].

From a practical perspective, these results highlight the potential of EMA as a core measurement component in digital mental health monitoring and intervention systems. Because EMA of emotional exhaustion can capture both sustained changes in mean symptom levels and short-term person-specific fluctuations, it is particularly well suited to support just-in-time adaptive interventions (JITAIs), where timely detection of within-person change is essential for triggering and providing support at the right moment. At the same time, the external validity of these implications is constrained by the characteristics of the present sample. As such, the applicability of EMA-based monitoring to broader occupational populations should be evaluated cautiously. Retrospective measurements, by contrast, are more limited in their added value for JITAIs because they summarize experiences only after some time has passed and primarily reflect aggregated components of symptom severity.

More generally, the present findings demonstrate that the choice of assessment strategy should be guided by the temporal dynamics of the target process rather than by feasibility or tradition alone. When the research or clinical goal is to diagnose or understand stable differences between individuals at specific points in time, retrospective questionnaires may be sufficient. However, when the goal is to understand within-person dynamics, early warning signals or mechanisms of change, intensive longitudinal approaches such as EMA are methodologically necessary and, based on the results of the current study, feasible to implement during work reintegration.

### Limitations

Several limitations should nevertheless be acknowledged. First, the small sample (N=18) limits generalizability and statistical power, especially for detecting more subtle effects. However, the intensive longitudinal design resulted in a large number of repeated observations per participant, making the data well-suited for idiographic and within-person process analysis [35]. Participants were also part of a specific clinical subgroup: individuals on long-term sick leave due to stress- or burnout-related symptoms who were entering a clinician-guided return-to-work trajectory. Their symptom severity, recovery patterns, and occupational circumstances may differ substantially from those of the broader working population or from individuals experiencing milder or more heterogeneous forms of burnout. As a result, the extent to which the present findings generalize to other occupational groups, industries, or reintegration contexts is uncertain. At the same time, this population is still relatively underrepresented in empirical research and the present study provides initial within-person evidence from a group for whom detailed temporal data are rarely available. These findings therefore offer an important first step toward understanding daily symptom dynamics during occupational rehabilitation, even if broader generalization requires further work in larger and more diverse samples. Second, the intensive assessment schedule could have increased participant burden and potentially affected data completeness. Nevertheless, participants completed 87.82% (n=3157) of the scheduled daily EMA assessments. This level of adherence is comparable to that reported by Berkemeier et al [47] of 88.25%, demonstrating that high compliance can be maintained over a 9-month EMA study involving 16 PhD candidates. Furthermore, the EMA adherence observed in the present study exceeded the mean compliance rate of approximately 79% reported across 477 EMA studies, most of which had substantially shorter assessment periods [48]. Adherence to the monthly retrospective BAT questionnaires was somewhat lower (n=94, 76.4%). Yet, it remained comparable to the study of Jongen et al [49] who reported a response rate of 87.1% after 6 months and 75.5% after 12 months of monthly online assessments among individuals with multiple sclerosis. Considering that participants in the present study were recovering from burnout and could experience fatigue and cognitive difficulties that may interfere with repeated questionnaire completion, these findings suggest that combining brief daily EMA with monthly retrospective assessments can be feasible during return-to-work trajectories. Third, only one EMA construct (daily emotional exhaustion) was examined, while other affective or stress-related states may show different patterns. Fourth, the study focused on monthly questionnaires. Shorter recall periods (eg, weekly) may be more sensitive to variability and short-term dynamics [50,51]. Finally, although nonlinear time trends were modeled, the analyses remain descriptive and do not support causal inference.

### Future Directions

Future research should examine whether similar EMA-questionnaire correspondence patterns emerge in more heterogeneous occupational groups, including employees with varying symptom severity, job types, and reintegration contexts. Integrating EMA with multimodal and passive sensing data streams, such as smartphone and wearable-derived indicators of behavior, physiology, and context, may further clarify whether these findings generalize across settings and whether EMA-derived indicators can reliably detect clinically relevant transitions such as relapse or sustained improvement. Once sufficiently validated, this could even enable the partial replacement of EMA measures with more continuous, unobtrusive, and low-burden measures, particularly in preventive and rehabilitation contexts. Finally, future research in larger and more diverse samples could examine whether the intensity or timing of occupational-physician counseling sessions interacts with EMA-based symptom dynamics or monthly burnout trajectories. The present sample was too small to support meaningful comparisons across session counts, but such work may clarify how reintegration support relates to within-person change during recovery.

### Conclusions

This study showed that daily emotional exhaustion can capture within-person changes also reflected in monthly burnout symptom reports during a 6-month work reintegration period, particularly Exhaustion and Cognitive Impairment, whereas day-to-day variability did not add explanatory value beyond mean levels. These findings suggest that the choice of assessment strategy should be guided by the research’s temporal and clinical goals. Retrospective questionnaires may be suitable for assessing overall symptom severity at specific time points, where EMA can provide additional insight into within-person dynamics and changes over time. This suggests that EMA may be particularly suitable for longitudinal monitoring in restorative contexts, such as work reintegration, where the goal is to follow individual trajectories over time and detect meaningful changes or risk periods. When the goal is a diagnosis or more global overview of differences in symptom levels across individuals at specific time points, retrospective questionnaires may still be more appropriate. More broadly, the present findings lay an initial foundation for the development of clinically relevant JITAIs that use longitudinal monitoring to detect meaningful changes in burnout symptoms and provide timely, personalized support during work reintegration.

## Supplementary material

10.2196/92458 Multimedia Appendix 1 Complete R Markdown workflow documenting all analyses conducted in the study, including data preprocessing, data preparation and transformation, statistical analyses, and associated analytical procedures.

## Abbreviations

BAT: Burnout Assessment Tool
EMA: ecological momentary assessment
ICC: intraclass correlation coefficient
JITAI: just-in-time adaptive intervention
