# Supplementary material for: Associations Between Daily EMA of Emotional Exhaustion and Monthly Burnout Symptoms During Work Reintegration: Longitudinal Multilevel Study
Source: JMIR Form Res. 2026 Oct 13;10:e92458. doi: 10.2196/92458 (PMC13660457; doi:10.2196/92458)
Supplement: Supplementary file 1 [file formative-v10-e92458-s001.docx]

Supplemental materials

S1. R Markdown

R Markdown Berkemeier et al

2026-07-27

## R Markdown

# Preprocessing

```
#DF monthly
df_monthly <- df_monthly[,-c(3, 5, 7, 9, 11, 13, 15, 17, 19, 21, 23, 25, 27, 29, 31, 33, 35, 37, 39, 41, 43, 45, 47, 49, 51, 53, 55, 57, 59, 61, 63, 65, 67, 69, 71, 73, 75, 77, 79, 81, 83, 85, 87, 89, 91, 93, 95, 97, 99, 101, 103, 105, 107, 109, 111, 113, 115, 117, 119, 121, 123, 125, 127, 129, 131, 133, 135, 137, 139, 141, 143, 147, 149, 151, 153, 154, 156:168, 170:174, 176)]
df_monthly$month <- format(as.Date(df_monthly$Record.Time, format="%Y-%m-%d"),"%m")
df_monthly$date <- format(as.Date(df_monthly$Record.Time, format="%Y-%m-%d"),"%Y-%m-%d")
df_monthly <- df_monthly %>%
  dplyr::rename(
    BAT_SF_1 = X.20_SAQ..Werkbeleving ,
    BAT_SF_2 = X.21_SAQ..Werkbeleving,
    BAT_SF_3 = X.22_SAQ..Werkbeleving,
    BAT_SF_4 = X.23_SAQ..Werkbeleving,
    BAT_SF_5 = X.24_SAQ..Werkbeleving,
    BAT_SF_6 = X.25_SAQ..Werkbeleving,
    BAT_SF_7 = X.26_SAQ..Werkbeleving,
    BAT_SF_8 = X.27_SAQ..Werkbeleving,
    BAT_SF_9 = X.28_SAQ..Werkbeleving,
    BAT_SF_10 = X.29_SAQ..Werkbeleving,
    BAT_SF_11 = X.30_SAQ..Werkbeleving,
    BAT_SF_12 = X.31_SAQ..Werkbeleving,
    Work_situation = X.83_SAQ..Werksituatie,
    person = Participant.ID,
    Duration = Duration..seconds..from.first.response.to.completion.time
  )

rep_str = c('107504'='re1',
            '107505'='re2',
            '107506'='re3',
            '107507'='re4',
            '107508'='re5',
            '107509'='re6',
            '107510'='re7',
            '107511'='re8',
            '107512'='re9',
            '107513'='re10',
            '107514'='re11',
            '107515'='re12',
            '107516'='re13',
            '107517'='re14',
            '107518'='re15',
            '107519'='re16',
            '107520'='re17',
            '107521'='re18',
            '107522'='re19')
df_monthly <- df_monthly[!(df_monthly$person %in% c('105814', '107563')), ]
df_monthly$person <- str_replace_all(df_monthly$person, rep_str)

df_monthly <- df_monthly %>%
  dplyr::select(date, person, month, Work_situation, BAT_SF_1, BAT_SF_2, BAT_SF_3, BAT_SF_4,
                BAT_SF_5, BAT_SF_6, BAT_SF_7, BAT_SF_8, BAT_SF_9, BAT_SF_10, BAT_SF_11, BAT_SF_12, Duration) %>%
  drop_na(date)


#Recode BAT items (scoring)
bat_items <- c("BAT_SF_1", "BAT_SF_2", "BAT_SF_3", "BAT_SF_4", "BAT_SF_5", "BAT_SF_6", "BAT_SF_7", "BAT_SF_8", "BAT_SF_9", "BAT_SF_10", "BAT_SF_11", "BAT_SF_12")
recode_bat <- function(x) {
  ifelse(x == "Nooit", 1,
         ifelse(x == "Zelden", 2,
                ifelse(x == "Soms", 3,
                       ifelse(x == "Vaak", 4,
                              ifelse(x == "Altijd", 5, x)))))
}
df_monthly[bat_items] <- lapply(df_monthly[bat_items], recode_bat)

exclude_ids_recoded <- c("re12")

df_monthly <- df_monthly %>% filter(!(person %in% exclude_ids_recoded))

# Compute total scores per questionnaire
# First, convert all relevant questionnaire columns to numeric
df_monthly_score <- df_monthly %>%
  mutate(across(matches("^(BAT_)"), ~ as.numeric(as.character(.)))) %>%
  mutate(
    BAT_Exhaustion = BAT_SF_1 + BAT_SF_2 + BAT_SF_3,
    BAT_Mental_distance = BAT_SF_4 + BAT_SF_5 + BAT_SF_6,
    BAT_Cognitive_impairment = BAT_SF_7 + BAT_SF_8 + BAT_SF_9,
    BAT_Emotional_impairment = BAT_SF_10 + BAT_SF_11 + BAT_SF_12
  )

df_monthly_score <- df_monthly_score %>%
  mutate(
    date = as.Date(date),
    person = as.factor(person)
  )

# Figure 3 subscales over time

ids <- sort(unique(df_monthly_score$person))
color_map <- setNames(scales::hue_pal()(length(ids)), ids)

bat_vars <- c("BAT_Exhaustion","BAT_Mental_distance","BAT_Cognitive_impairment","BAT_Emotional_impairment")

df_bat_long <- df_monthly_score %>%
  select(person, date, all_of(bat_vars)) %>%
  pivot_longer(cols = all_of(bat_vars), names_to = "subscale", values_to = "score") %>%
  drop_na(score)

p_overall <- ggplot(df_bat_long, aes(x = date, y = score, color = person)) +
  geom_point(alpha = 0.25, size = 1) +
  geom_smooth(method = "loess", se = FALSE, linewidth = 1) +
  facet_wrap(~ subscale, scales = "free_y") +
  scale_color_manual(values = color_map) +
  scale_x_date(
    date_breaks = "1 month",
    labels = label_date(format = "%b %Y", locale = "en"),
    expand = c(0, 0)
  ) +
  labs(
    title = "BAT subscales over time (overall + person-specific trends)",
    x = "Date",
    y = "BAT score"
  ) +
  theme_minimal(base_size = 12) +
  theme(
    axis.text.x = element_text(angle = 45, hjust = 1),
    legend.position = "bottom"
  )

print(p_overall)
## `geom_smooth()` using formula = 'y ~ x'
```

```
#DF EMA Emotional Exhaustion
df_emo_exh <- df_emo_exh[,-c(3, 4, 6:18, 20:26)]
df_emo_exh <- dplyr::rename(df_emo_exh, person = Participant.ID)
df_emo_exh <- dplyr::rename(df_emo_exh, emotional_exhaustion = Emotionele_uitputting)
df_emo_exh$month <- format(as.Date(df_emo_exh$Record.Time, format="%Y-%m-%d"),"%m")
df_emo_exh$date <- format(as.Date(df_emo_exh$Record.Time, format="%Y-%m-%d"),"%Y-%m-%d")
rep_str = c('107504'='re1',
            '107505'='re2',
            '107506'='re3',
            '107507'='re4',
            '107508'='re5',
            '107509'='re6',
            '107510'='re7',
            '107511'='re8',
            '107512'='re9',
            '107513'='re10',
            '107514'='re11',
            '107515'='re12',
            '107516'='re13',
            '107517'='re14',
            '107518'='re15',
            '107519'='re16',
            '107520'='re17',
            '107521'='re18',
            '107522'='re19')
df_emo_exh <- df_emo_exh[!(df_emo_exh$person %in% c('105814', '107563')), ]
df_emo_exh$person <- str_replace_all(df_emo_exh$person, rep_str)

df_emo_exh <- df_emo_exh %>% filter(!(person %in% exclude_ids_recoded))

df_emo_exh <- df_emo_exh %>%
  drop_na(date)

#Figure 1
# 1) Ensure person order
df_emo_exh$person <- factor(df_emo_exh$person, levels = paste0("re", 1:19))

# 2) Define a consistent color map (ggplot default hue palette)
color_map <- scales::hue_pal()(19)
names(color_map) <- paste0("re", 1:19)

df_emo_exh$date <- as.Date(df_emo_exh$date)

ggplot(df_emo_exh, aes(x = date, y = emotional_exhaustion, color = person)) +
  geom_point() +
  geom_smooth(aes(group = person), method = "loess", se = FALSE, color = "black") +
  facet_wrap(~ person) +
  scale_color_manual(values = color_map, guide = "none") +
  scale_x_date(
    date_breaks = "1 month",
    labels = scales::label_date(format = "%b %Y", locale = "en"),  # ← force English
    expand      = c(0, 0)
  ) +
  labs(
    title = "Trend of Emotional Exhaustion per Person",
    x     = "Month",
    y     = "Emotional Exhaustion"
  ) +
  theme_minimal() +
  theme(axis.text.x = element_text(angle = 45, hjust = 1))
## `geom_smooth()` using formula = 'y ~ x'
```

## Preparation analyses

```
# -----------------------------
# 0) Ensure dates + ordering
# -----------------------------
df_monthly_score <- df_monthly_score %>%
  mutate(
    person = as.character(person),
    date  = as.Date(date),
    month = as.numeric(month)
  ) %>%
  arrange(person, date) %>%
  distinct(person, date, .keep_all = TRUE)

df_emo_exh <- df_emo_exh %>%
  mutate(date = as.Date(date)) %>%
  arrange(person, date)

# ----------------------------------------------------------
# 1) DEFINE OUTCOMES (monthly questionnaires)
# ----------------------------------------------------------
outcomes_raw <- c(
  "BAT_Exhaustion",
  "BAT_Mental_distance",
  "BAT_Cognitive_impairment",
  "BAT_Emotional_impairment"
)

df_y <- df_monthly_score %>%
  select(person, date, month, all_of(outcomes_raw)) %>%
  distinct(person, date, .keep_all = TRUE) %>%
  arrange(person, date)

# ----------------------------------------------------------
# 2) EMA FEATURE ENGINEERING (mean + SD ONLY)
# ----------------------------------------------------------
window_days <- 30
min_ema_n   <- 5   # minimum EMA obs in window for mean/SD

compute_ema_mean_sd <- function(pid, qd, ema_df, window_days = 30, min_n = 5) {
  qd <- as.Date(qd)
  w_start <- qd - window_days

  W <- ema_df %>%
    filter(person == pid, date >= w_start, date < qd) %>%
    arrange(date) %>%
    drop_na(emotional_exhaustion)

  n_obs <- nrow(W)

  if (n_obs < min_n) {
    return(tibble(
      person = pid, date = qd,
      ema_n = n_obs,
      exh_mean = NA_real_,
      exh_sd   = NA_real_
    ))
  }

  tibble(
    person = pid, date = qd,
    ema_n = n_obs,
    exh_mean = mean(W$emotional_exhaustion, na.rm = TRUE),
    exh_sd   = sd(W$emotional_exhaustion,   na.rm = TRUE)
  )
}

ema_features <- df_y %>%
  distinct(person, date) %>%
  rowwise() %>%
  do(compute_ema_mean_sd(
    pid = .$person,
    qd  = .$date,
    ema_df = df_emo_exh,
    window_days = window_days,
    min_n = min_ema_n
  )) %>%
  ungroup()

# -----------------------------
# 3) Join + construct df_model
# -----------------------------
df_y <- df_y %>%
  mutate(date = as.Date(date),
         person = as.character(person))

ema_features <- ema_features %>%
  mutate(date = as.Date(date),
         person = as.character(person))

df_model <- df_y %>%
  left_join(ema_features, by = c("person", "date")) %>%
  filter(!is.na(exh_mean), !is.na(exh_sd)) %>%
  arrange(person, date) %>%
  group_by(person) %>%
  mutate(time = as.numeric(date - min(date, na.rm = TRUE))) %>%  # days since first questionnaire
  ungroup() %>%
  mutate(
    time_c = as.numeric(scale(time, center = TRUE, scale = FALSE)),
    time_z = as.numeric(scale(time))
  ) %>%
  group_by(person) %>%
  mutate(
    exh_mean_bp = mean(exh_mean, na.rm = TRUE),
    exh_mean_wp = exh_mean - exh_mean_bp
  ) %>%
  ungroup()

# ----------------------------------------------------------
# 4) Descriptives + correlations (EMA predictors vs outcomes)
# ----------------------------------------------------------

# Desired order + labels for outcomes
outcomes <- c(
  "BAT_Exhaustion",
  "BAT_Cognitive_impairment",
  "BAT_Emotional_impairment",
  "BAT_Mental_distance"
)

# Predictors (with desired label order top-to-bottom)
predictors <- c("exh_mean_wp", "exh_sd")

# Label maps
pred_labs <- c(
  exh_mean_wp = "Within-person mean",
  exh_sd      = "Variability (SD)"
)

out_labs <- c(
  BAT_Exhaustion            = "Exhaustion",
  BAT_Cognitive_impairment  = "Cognitive Impairment",
  BAT_Emotional_impairment  = "Emotional Impairment",
  BAT_Mental_distance       = "Mental Distance"
)

corr_df <- expand.grid(
  predictor = predictors,
  outcome   = outcomes,
  stringsAsFactors = FALSE
) %>%
  rowwise() %>%
  mutate(
    r = cor(df_model[[predictor]], df_model[[outcome]], use = "pairwise.complete.obs"),
    p = tryCatch(cor.test(df_model[[predictor]], df_model[[outcome]])$p.value,
                 error = function(e) NA_real_)
  ) %>%
  ungroup() %>%
  mutate(
    sig = case_when(
      !is.na(p) & p < .001 ~ "***",
      !is.na(p) & p < .01  ~ "**",
      !is.na(p) & p < .05  ~ "*",
      TRUE ~ ""
    ),
    # apply readable labels
    predictor_lab = recode(predictor, !!!pred_labs),
    outcome_lab   = recode(outcome, !!!out_labs),
    # enforce plotting order
    outcome_lab   = factor(outcome_lab, levels = unname(out_labs)),
    predictor_lab = factor(predictor_lab, levels = c("Within-person mean", "Variability (SD)"))
  )

print(corr_df)
## # A tibble: 8 × 7
##   predictor   outcome                   r      p sig   predictor_lab outcome_lab
##   <chr>       <chr>                 <dbl>  <dbl> <chr> <fct>         <fct>
## 1 exh_mean_wp BAT_Exhaustion      0.215   0.0370 "*"   Within-perso… Exhaustion
## 2 exh_sd      BAT_Exhaustion      0.00279 0.979  ""    Variability … Exhaustion
## 3 exh_mean_wp BAT_Cognitive_imp…  0.205   0.0472 "*"   Within-perso… Cognitive …
## 4 exh_sd      BAT_Cognitive_imp… -0.124   0.232  ""    Variability … Cognitive …
## 5 exh_mean_wp BAT_Emotional_imp…  0.110   0.292  ""    Within-perso… Emotional …
## 6 exh_sd      BAT_Emotional_imp…  0.0750  0.473  ""    Variability … Emotional …
## 7 exh_mean_wp BAT_Mental_distan…  0.157   0.131  ""    Within-perso… Mental Dis…
## 8 exh_sd      BAT_Mental_distan… -0.0142  0.892  ""    Variability … Mental Dis…
ggplot(corr_df, aes(x = outcome_lab, y = predictor_lab, fill = r)) +
  geom_tile(color = "white") +
  scale_fill_gradient2(
    low = "#B2182B", mid = "white", high = "#2166AC",
    midpoint = 0, limits = c(-1, 1), name = "r"
  ) +
  geom_text(aes(label = paste0(sprintf("%.2f", r), sig)),
            size = 6, fontface = "bold") +
  theme_minimal(base_size = 12) +
  theme(
    axis.text.x = element_text(angle = 45, hjust = 1, size = 12),
    axis.text.y = element_text(size = 12),
    panel.grid  = element_blank(),
    legend.position = "right"
  ) +
  labs(
    title = "Correlations Between EMA Predictors and BAT Subscales",
    x = "BAT subscale",
    y = "EMA predictor"
  )
```

# Multilevel models

```
# ----------------------------------------------------------
# 5) Fit multilevel models
# ----------------------------------------------------------
#Cognitive Impairment
m_ci <- lmer(
  BAT_Cognitive_impairment ~
    time_c +
    exh_mean_wp + exh_mean_bp + exh_sd +
    (1 | person),
  data = df_model,
  REML = FALSE
)
summary(m_ci)
## Linear mixed model fit by maximum likelihood . t-tests use Satterthwaite's
##   method [lmerModLmerTest]
## Formula: BAT_Cognitive_impairment ~ time_c + exh_mean_wp + exh_mean_bp +
##     exh_sd + (1 | person)
##    Data: df_model
##
##       AIC       BIC    logLik -2*log(L)  df.resid
##     367.1     384.9    -176.5     353.1        87
##
## Scaled residuals:
##      Min       1Q   Median       3Q      Max
## -2.13979 -0.51723 -0.09304  0.55807  2.65535
##
## Random effects:
##  Groups   Name        Variance Std.Dev.
##  person   (Intercept) 4.619    2.149
##  Residual             1.448    1.203
## Number of obs: 94, groups:  person, 18
##
## Fixed effects:
##              Estimate Std. Error        df t value Pr(>|t|)
## (Intercept)  7.878123   1.401129 20.337507   5.623 1.57e-05 ***
## time_c      -0.007100   0.002959 79.395379  -2.400   0.0188 *
## exh_mean_wp  0.041320   0.020134 77.896786   2.052   0.0435 *
## exh_mean_bp  0.004777   0.028425 17.768622   0.168   0.8684
## exh_sd      -0.014704   0.037202 92.866593  -0.395   0.6936
## ---
## Signif. codes:  0 '***' 0.001 '**' 0.01 '*' 0.05 '.' 0.1 ' ' 1
##
## Correlation of Fixed Effects:
##             (Intr) time_c exh_mn_w exh_mn_b
## time_c      -0.095
## exh_mean_wp  0.080  0.399
## exh_mean_bp -0.862 -0.005  0.007
## exh_sd      -0.288  0.296 -0.244   -0.063
confint(m_ci, method = "profile")
## Computing profile confidence intervals ...
##                    2.5 %       97.5 %
## .sig01       1.555345316  3.156029177
## .sigma       1.034425516  1.423648959
## (Intercept)  4.961669310 10.730649094
## time_c      -0.013000439 -0.001241121
## exh_mean_wp  0.001429803  0.081381796
## exh_mean_bp -0.054150803  0.063567041
## exh_sd      -0.089290277  0.059495056
performance::check_model(m_ci)
print(performance::r2_nakagawa(m_ci))
## # R2 for Mixed Models
##
##   Conditional R2: 0.775
##      Marginal R2: 0.056
print(performance::check_collinearity(m_ci))
## # Check for Multicollinearity
##
## Low Correlation
##
##         Term  VIF       VIF 95% CI adj. VIF Tolerance Tolerance 95% CI
##       time_c 1.48 [1.22,     2.02]     1.22      0.68     [0.49, 0.82]
##  exh_mean_wp 1.43 [1.19,     1.97]     1.20      0.70     [0.51, 0.84]
##  exh_mean_bp 1.00 [1.00, 2.25e+15]     1.00      1.00     [0.00, 1.00]
##       exh_sd 1.33 [1.13,     1.84]     1.15      0.75     [0.54, 0.89]
#Emotional Impairment
m_ei <- lmer(
  BAT_Emotional_impairment ~
    time_c +
    exh_mean_wp + exh_mean_bp + exh_sd +
    (1 | person),
  data = df_model,
  REML = FALSE
)
summary(m_ei)
## Linear mixed model fit by maximum likelihood . t-tests use Satterthwaite's
##   method [lmerModLmerTest]
## Formula: BAT_Emotional_impairment ~ time_c + exh_mean_wp + exh_mean_bp +
##     exh_sd + (1 | person)
##    Data: df_model
##
##       AIC       BIC    logLik -2*log(L)  df.resid
##     378.9     396.7    -182.5     364.9        87
##
## Scaled residuals:
##      Min       1Q   Median       3Q      Max
## -2.13591 -0.63886 -0.00411  0.58036  2.63651
##
## Random effects:
##  Groups   Name        Variance Std.Dev.
##  person   (Intercept) 4.644    2.155
##  Residual             1.687    1.299
## Number of obs: 94, groups:  person, 18
##
## Fixed effects:
##              Estimate Std. Error        df t value Pr(>|t|)
## (Intercept)  6.525616   1.418065 20.807398   4.602 0.000158 ***
## time_c      -0.015756   0.003189 79.964876  -4.941 4.19e-06 ***
## exh_mean_wp -0.008533   0.021713 78.336196  -0.393 0.695407
## exh_mean_bp  0.024174   0.028633 17.976542   0.844 0.409606
## exh_sd      -0.024451   0.039725 93.622582  -0.616 0.539708
## ---
## Signif. codes:  0 '***' 0.001 '**' 0.01 '*' 0.05 '.' 0.1 ' ' 1
##
## Correlation of Fixed Effects:
##             (Intr) time_c exh_mn_w exh_mn_b
## time_c      -0.099
## exh_mean_wp  0.084  0.401
## exh_mean_bp -0.855 -0.004  0.007
## exh_sd      -0.304  0.292 -0.241   -0.067
confint(m_ei, method = "profile")
## Computing profile confidence intervals ...
##                   2.5 %       97.5 %
## .sig01       1.55656827  3.166717361
## .sigma       1.11692012  1.536488160
## (Intercept)  3.61909993  9.448157709
## time_c      -0.02207795 -0.009426882
## exh_mean_wp -0.05169172  0.034499872
## exh_mean_bp -0.03499973  0.083509218
## exh_sd      -0.10315982  0.054320606
performance::check_model(m_ei)
print(performance::r2_nakagawa(m_ei))
## # R2 for Mixed Models
##
##   Conditional R2: 0.766
##      Marginal R2: 0.121
print(performance::check_collinearity(m_ei))
## # Check for Multicollinearity
##
## Low Correlation
##
##         Term  VIF       VIF 95% CI adj. VIF Tolerance Tolerance 95% CI
##       time_c 1.47 [1.22,     2.02]     1.21      0.68     [0.50, 0.82]
##  exh_mean_wp 1.43 [1.19,     1.96]     1.20      0.70     [0.51, 0.84]
##  exh_mean_bp 1.01 [1.00, 2.12e+13]     1.00      0.99     [0.00, 1.00]
##       exh_sd 1.32 [1.12,     1.83]     1.15      0.76     [0.55, 0.89]
#Mental Distance
m_md <- lmer(
  BAT_Mental_distance ~
    time_c +
    exh_mean_wp + exh_mean_bp + exh_sd +
    (1 | person),
  data = df_model,
  REML = FALSE
)
summary(m_md)
## Linear mixed model fit by maximum likelihood . t-tests use Satterthwaite's
##   method [lmerModLmerTest]
## Formula: BAT_Mental_distance ~ time_c + exh_mean_wp + exh_mean_bp + exh_sd +
##     (1 | person)
##    Data: df_model
##
##       AIC       BIC    logLik -2*log(L)  df.resid
##     401.1     418.9    -193.5     387.1        87
##
## Scaled residuals:
##      Min       1Q   Median       3Q      Max
## -3.03149 -0.55340 -0.00506  0.59437  2.09468
##
## Random effects:
##  Groups   Name        Variance Std.Dev.
##  person   (Intercept) 3.829    1.957
##  Residual             2.338    1.529
## Number of obs: 94, groups:  person, 18
##
## Fixed effects:
##              Estimate Std. Error        df t value Pr(>|t|)
## (Intercept)  8.639464   1.341747 21.554221   6.439 1.95e-06 ***
## time_c      -0.015231   0.003725 81.463693  -4.089 0.000101 ***
## exh_mean_wp  0.002945   0.025446 79.324109   0.116 0.908166
## exh_mean_bp -0.023814   0.026581 17.937867  -0.896 0.382159
## exh_sd       0.015589   0.044569 92.487986   0.350 0.727312
## ---
## Signif. codes:  0 '***' 0.001 '**' 0.01 '*' 0.05 '.' 0.1 ' ' 1
##
## Correlation of Fixed Effects:
##             (Intr) time_c exh_mn_w exh_mn_b
## time_c      -0.111
## exh_mean_wp  0.096  0.412
## exh_mean_bp -0.827 -0.003  0.007
## exh_sd      -0.359  0.274 -0.230   -0.083
confint(m_md, method = "profile")
## Computing profile confidence intervals ...
##                   2.5 %       97.5 %
## .sig01       1.38219353  2.911097389
## .sigma       1.31482410  1.808781778
## (Intercept)  5.85499122 11.364599851
## time_c      -0.02263473 -0.007851776
## exh_mean_wp -0.04748005  0.053512816
## exh_mean_bp -0.07888356  0.031143892
## exh_sd      -0.07300871  0.104496298
performance::check_model(m_md)
print(performance::r2_nakagawa(m_md))
## # R2 for Mixed Models
##
##   Conditional R2: 0.667
##      Marginal R2: 0.121
print(performance::check_collinearity(m_md))
## # Check for Multicollinearity
##
## Low Correlation
##
##         Term  VIF       VIF 95% CI adj. VIF Tolerance Tolerance 95% CI
##       time_c 1.46 [1.21,     2.00]     1.21      0.69     [0.50, 0.83]
##  exh_mean_wp 1.42 [1.19,     1.95]     1.19      0.70     [0.51, 0.84]
##  exh_mean_bp 1.01 [1.00, 1.72e+08]     1.00      0.99     [0.00, 1.00]
##       exh_sd 1.29 [1.10,     1.80]     1.13      0.78     [0.56, 0.91]
#Emotional Exhaustion
# Spline time for BAT_Exhaustion
m_exh <- lmer(
  BAT_Exhaustion ~
    ns(time_z, df = 3) +
    exh_mean_wp + exh_mean_bp + exh_sd +
    (1 | person),
  data = df_model,
  REML = FALSE
)
summary(m_exh)
## Linear mixed model fit by maximum likelihood . t-tests use Satterthwaite's
##   method [lmerModLmerTest]
## Formula: BAT_Exhaustion ~ ns(time_z, df = 3) + exh_mean_wp + exh_mean_bp +
##     exh_sd + (1 | person)
##    Data: df_model
##
##       AIC       BIC    logLik -2*log(L)  df.resid
##     379.7     402.6    -180.8     361.7        85
##
## Scaled residuals:
##      Min       1Q   Median       3Q      Max
## -2.45601 -0.41504  0.01391  0.46553  2.02251
##
## Random effects:
##  Groups   Name        Variance Std.Dev.
##  person   (Intercept) 4.998    2.236
##  Residual             1.592    1.262
## Number of obs: 94, groups:  person, 18
##
## Fixed effects:
##                     Estimate Std. Error       df t value Pr(>|t|)
## (Intercept)          8.77796    1.55197 25.15655   5.656 6.75e-06 ***
## ns(time_z, df = 3)1 -0.91890    0.64013 77.49248  -1.435 0.155171
## ns(time_z, df = 3)2  1.60412    1.08163 81.18783   1.483 0.141933
## ns(time_z, df = 3)3 -0.47760    0.65116 77.92991  -0.733 0.465483
## exh_mean_wp          0.07283    0.02123 78.16157   3.430 0.000965 ***
## exh_mean_bp         -0.01146    0.02963 18.06348  -0.387 0.703305
## exh_sd               0.01845    0.04160 93.78892   0.443 0.658466
## ---
## Signif. codes:  0 '***' 0.001 '**' 0.01 '*' 0.05 '.' 0.1 ' ' 1
##
## Correlation of Fixed Effects:
##             (Intr) n(_,d=3)1 n(_,d=3)2 n(_,d=3)3 exh_mn_w exh_mn_b
## ns(t_,d=3)1 -0.116
## ns(t_,d=3)2 -0.321 -0.083
## ns(t_,d=3)3 -0.113 -0.207     0.514
## exh_mean_wp  0.010  0.100     0.287     0.351
## exh_mean_bp -0.795 -0.025    -0.025     0.027     0.009
## exh_sd      -0.383  0.220     0.354     0.049    -0.233   -0.079
confint(m_exh, method = "profile")
## Computing profile confidence intervals ...
##                           2.5 %      97.5 %
## .sig01               1.62118138  3.27853048
## .sigma               1.08484964  1.49240363
## (Intercept)          5.58173509 11.91541290
## ns(time_z, df = 3)1 -2.19236574  0.34948430
## ns(time_z, df = 3)2 -0.54922564  3.74604228
## ns(time_z, df = 3)3 -1.77185874  0.81302920
## exh_mean_wp          0.03072446  0.11498582
## exh_mean_bp         -0.07283482  0.04976373
## exh_sd              -0.06433699  0.10111673
performance::check_model(m_exh)
print(performance::r2_nakagawa(m_exh))
## # R2 for Mixed Models
##
##   Conditional R2: 0.777
##      Marginal R2: 0.075
print(performance::check_collinearity(m_exh))
## # Check for Multicollinearity
##
## Low Correlation
##
##                Term  VIF       VIF 95% CI adj. VIF Tolerance Tolerance 95% CI
##  ns(time_z, df = 3) 1.71 [1.38,     2.33]     1.31      0.58     [0.43, 0.72]
##         exh_mean_wp 1.45 [1.21,     1.97]     1.20      0.69     [0.51, 0.83]
##         exh_mean_bp 1.01 [1.00, 1.34e+08]     1.00      0.99     [0.00, 1.00]
##              exh_sd 1.51 [1.25,     2.05]     1.23      0.66     [0.49, 0.80]
############################################################
# FOREST PLOT
############################################################
bat_colors <- c(
  "BAT: Exhaustion" = "#0072B2",
  "BAT: Mental distance" = "#D55E00",
  "BAT: Cognitive impairment" = "#009E73",
  "BAT: Emotional impairment" = "#CC79A7"
)

outcome_labs <- c(
  BAT_Exhaustion = "BAT: Exhaustion",
  BAT_Mental_distance = "BAT: Mental distance",
  BAT_Cognitive_impairment = "BAT: Cognitive impairment",
  BAT_Emotional_impairment = "BAT: Emotional impairment"
)

mods <- list(
  BAT_Exhaustion = m_exh,
  BAT_Mental_distance = m_md,
  BAT_Cognitive_impairment = m_ci,
  BAT_Emotional_impairment = m_ei
)

coef_df <- imap_dfr(mods, ~{
  broom.mixed::tidy(.x, effects = "fixed", conf.int = TRUE, conf.method = "Wald") %>%
    mutate(outcome = .y)
}) %>%
  filter(term %in% c("exh_mean_wp", "exh_mean_bp", "exh_sd")) %>%
  mutate(
    term_label = recode(
      term,
      exh_mean_wp  = "Mean (within-person)",
      exh_mean_bp  = "Mean (between-person)",
      exh_sd       = "SD (window-based)"
    ),
    outcome_label = recode(outcome, !!!outcome_labs),
    sig = case_when(
      p.value < 0.001 ~ "***",
      p.value < 0.01  ~ "**",
      p.value < 0.05  ~ "*",
      p.value < 0.10  ~ "·",
      TRUE ~ ""
    )
  )

coef_df$outcome_label <- factor(coef_df$outcome_label, levels = unname(outcome_labs))
coef_df$term_label <- factor(
  coef_df$term_label,
  levels = c(
    "Mean (within-person)",
    "SD (window-based)",
    "Mean (between-person)"
  )
)

p_ema <- ggplot(coef_df, aes(x = estimate, y = outcome_label, color = outcome_label)) +
  geom_vline(xintercept = 0, linetype = 2, linewidth = 0.6, alpha = 0.7, color = "grey50") +
  geom_errorbar(aes(xmin = conf.low, xmax = conf.high), width = 0.18, linewidth = 0.7, alpha = 0.9) +
  geom_point(aes(shape = p.value < 0.05), size = 2.8) +
  geom_text(aes(label = sig), vjust = -0.9, size = 4, show.legend = FALSE) +
  facet_wrap(~ term_label, scales = "free_x") +
  scale_color_manual(values = bat_colors, name = "BAT subscale") +
  scale_shape_manual(values = c(`TRUE` = 16, `FALSE` = 1), guide = "none") +
  labs(
    title = "EMA predictors (fixed effects)",
    subtitle = "WP/BP mean + window SD predicting BAT subscales.",
    x = "β (fixed-effect estimate)",
    y = NULL
  ) +
  theme_minimal(base_size = 13) +
  theme(
    panel.grid.minor = element_blank(),
    strip.text = element_text(face = "bold"),
    plot.title = element_text(face = "bold"),
    legend.position = "bottom",
    axis.text.y = element_text(size = 11)
  )

print(p_ema)
```

# BAT internal consistency

```
items_exh <- c("BAT_SF_1", "BAT_SF_2", "BAT_SF_3")
items_md  <- c("BAT_SF_4", "BAT_SF_5", "BAT_SF_6")
items_ci  <- c("BAT_SF_7", "BAT_SF_8", "BAT_SF_9")
items_ei  <- c("BAT_SF_10", "BAT_SF_11", "BAT_SF_12")

alpha_exh <- psych::alpha(df_monthly_score[, items_exh])
alpha_md  <- psych::alpha(df_monthly_score[, items_md])
alpha_ci  <- psych::alpha(df_monthly_score[, items_ci])
alpha_ei  <- psych::alpha(df_monthly_score[, items_ei])

alphas <- tibble::tibble(
  Subscale = c("Exhaustion", "Mental Distance", "Cognitive Impairment", "Emotional Impairment"),
  Alpha = c(
    alpha_exh$total$raw_alpha,
    alpha_md$total$raw_alpha,
    alpha_ci$total$raw_alpha,
    alpha_ei$total$raw_alpha
  )
)

print(alphas)
## # A tibble: 4 × 2
##   Subscale             Alpha
##   <chr>                <dbl>
## 1 Exhaustion           0.852
## 2 Mental Distance      0.817
## 3 Cognitive Impairment 0.887
## 4 Emotional Impairment 0.922
```

#Comparison of mean and SD across two individuals

```
window_days <- 30
min_n <- 10

# Make sure dates are Date class
df_emo_exh <- df_emo_exh %>%
  mutate(date = as.Date(date))

df_monthly_dates <- df_monthly_score %>%
  transmute(
    person = as.character(person),
    date = as.Date(date)
  ) %>%
  distinct()

# Function that returns ONE row (tibble) or NULL
get_window <- function(pid, qd) {
  W <- df_emo_exh %>%
    filter(
      person == pid,
      date >= (qd - window_days),
      date < qd
    ) %>%
    select(person, date, emotional_exhaustion) %>%
    drop_na(emotional_exhaustion) %>%
    arrange(date)

  if (nrow(W) < min_n) return(NULL)

  tibble(
    person = pid,
    month_date = qd,
    n = nrow(W),
    mean_exh = mean(W$emotional_exhaustion),
    sd_exh   = sd(W$emotional_exhaustion),
    data = list(W)
  )
}

# Build window_list
window_list <- pmap_dfr(
  list(df_monthly_dates$person, df_monthly_dates$date),
  get_window
)

# Quick sanity check
print(nrow(window_list))
## [1] 121
print(head(window_list))
## # A tibble: 6 × 6
##   person month_date     n mean_exh sd_exh data
##   <chr>  <date>     <int>    <dbl>  <dbl> <list>
## 1 re1    2024-12-19    28     34.1  16.3  <df [28 × 3]>
## 2 re1    2025-01-18    25     23.2  18.4  <df [25 × 3]>
## 3 re1    2025-02-19    30     27.6  13.6  <df [30 × 3]>
## 4 re1    2025-03-18    30     23.0  12.0  <df [30 × 3]>
## 5 re1    2025-04-18    28     14.8   6.64 <df [28 × 3]>
## 6 re1    2025-05-18    30     18.6   6.38 <df [30 × 3]>
# ---- SETTINGS  ----
mean_tolerance <- 2      # max absolute difference in mean exhaustion
min_sd_ratio   <- 1.8    # SD must differ by at least this factor

# Find candidate pairs
pairs <- tidyr::crossing(
  i = seq_len(nrow(window_list)),
  j = seq_len(nrow(window_list))
) %>%
  filter(i < j) %>%
  mutate(
    pid_i = window_list$person[i],
    pid_j = window_list$person[j],
    date_i = window_list$month_date[i],
    date_j = window_list$month_date[j],
    mean_i = window_list$mean_exh[i],
    mean_j = window_list$mean_exh[j],
    sd_i   = window_list$sd_exh[i],
    sd_j   = window_list$sd_exh[j]
  ) %>%
  filter(pid_i != pid_j) %>%
  mutate(
    mean_diff = abs(mean_i - mean_j),
    sd_ratio  = pmax(sd_i, sd_j) / pmin(sd_i, sd_j)
  ) %>%
  filter(mean_diff <= mean_tolerance, sd_ratio >= min_sd_ratio) %>%
  arrange(mean_diff, desc(sd_ratio))

# Take best pair (or fail loudly if none)
stopifnot(nrow(pairs) > 0)

best <- pairs[1, ]
idx <- c(best$i, best$j)

example_windows <- window_list[idx, ] %>%
  mutate(example_id = paste0(person, " | ", as.character(month_date)))

plot_df <- example_windows %>%
  mutate(
    data = map(data, ~ .x %>%
                 select(date, emotional_exhaustion))
  ) %>%
  transmute(
    person,
    month_date,
    mean_exh,
    example_id,
    data
  ) %>%
  tidyr::unnest(data) %>%
  mutate(
    date = as.Date(date),
    person = as.factor(person),
    example_id = as.factor(example_id)
  )

ggplot(plot_df, aes(x = date, y = emotional_exhaustion, color = person, group = example_id)) +
  geom_line(linewidth = 0.9, alpha = 0.9) +
  geom_point(size = 1.8, alpha = 0.9) +
  geom_hline(
    data = example_windows,
    aes(yintercept = mean_exh, color = as.factor(person)),
    linetype = "dashed",
    linewidth = 0.9,
    inherit.aes = FALSE
  ) +
  facet_wrap(~ example_id, scales = "free_x") +
  scale_x_date(date_breaks = "1 week", date_labels = "%d %b") +
  labs(
    title = "Daily emotional exhaustion within selected person-month windows",
    subtitle = "Solid line = daily observations; dashed line = mean level within the window",
    x = "Date",
    y = "Emotional exhaustion (EMA)"
  ) +
  theme_minimal(base_size = 12) +
  theme(
    axis.text.x = element_text(angle = 45, hjust = 1),
    legend.position = "bottom"
  )
## Warning in geom_hline(data = example_windows, aes(yintercept = mean_exh, :
## Ignoring unknown parameters: `inherit.aes`
```

# ICC and descriptives

```
# Outcomes
bat_outcomes <- c(
  "BAT_Exhaustion",
  "BAT_Cognitive_impairment",
  "BAT_Emotional_impairment",
  "BAT_Mental_distance"
)

# Function to compute ICC from null model
compute_icc <- function(outcome, df) {

  fml <- as.formula(paste0(outcome, " ~ 1 + (1 | person)"))

  mod0 <- lmer(fml, data = df, REML = TRUE)

  icc <- performance::icc(mod0)

  tibble(
    outcome = outcome,
    ICC = icc$ICC_adjusted,
    ICC_unadjusted = icc$ICC_unadjusted
  )
}

# Run for all subscales
icc_table <- map_dfr(bat_outcomes, compute_icc, df = df_model)

print(icc_table)
## # A tibble: 4 × 3
##   outcome                    ICC ICC_unadjusted
##   <chr>                    <dbl>          <dbl>
## 1 BAT_Exhaustion           0.705          0.705
## 2 BAT_Cognitive_impairment 0.737          0.737
## 3 BAT_Emotional_impairment 0.684          0.684
## 4 BAT_Mental_distance      0.562          0.562
varcomp_table <- map_dfr(bat_outcomes, function(outcome) {

  fml <- as.formula(paste0(outcome, " ~ 1 + (1 | person)"))
  mod0 <- lmer(fml, data = df_model, REML = TRUE)

  vc <- as.data.frame(VarCorr(mod0))

  tibble(
    outcome = outcome,
    var_between = vc$vcov[vc$grp == "person"],
    var_within  = vc$vcov[vc$grp == "Residual"],
    ICC = vc$vcov[vc$grp == "person"] /
      (vc$vcov[vc$grp == "person"] + vc$vcov[vc$grp == "Residual"])
  )
})

print(varcomp_table)
## # A tibble: 4 × 4
##   outcome                  var_between var_within   ICC
##   <chr>                          <dbl>      <dbl> <dbl>
## 1 BAT_Exhaustion                  5.21       2.18 0.705
## 2 BAT_Cognitive_impairment        5.08       1.81 0.737
## 3 BAT_Emotional_impairment        5.10       2.36 0.684
## 4 BAT_Mental_distance             4.06       3.16 0.562
############################################################
# ICC FOR DAILY EMA EMOTIONAL EXHAUSTION
############################################################

# Make sure ID is factor
df_emo_exh$person <- as.factor(df_emo_exh$person)

# Random intercept-only model
icc_model <- lmer(
  emotional_exhaustion ~ 1 + (1 | person),
  data = df_emo_exh,
  REML = TRUE
)

summary(icc_model)
## Linear mixed model fit by REML. t-tests use Satterthwaite's method [
## lmerModLmerTest]
## Formula: emotional_exhaustion ~ 1 + (1 | person)
##    Data: df_emo_exh
##
## REML criterion at convergence: 26846.6
##
## Scaled residuals:
##     Min      1Q  Median      3Q     Max
## -3.2690 -0.6133 -0.0915  0.5558  3.9245
##
## Random effects:
##  Groups   Name        Variance Std.Dev.
##  person   (Intercept) 358.7    18.94
##  Residual             265.9    16.31
## Number of obs: 3177, groups:  person, 18
##
## Fixed effects:
##             Estimate Std. Error     df t value Pr(>|t|)
## (Intercept)   42.878      4.473 16.998   9.585 2.87e-08 ***
## ---
## Signif. codes:  0 '***' 0.001 '**' 0.01 '*' 0.05 '.' 0.1 ' ' 1
# Compute ICC
icc_res <- performance::icc(icc_model)
print(icc_res)
## # Intraclass Correlation Coefficient
##
##     Adjusted ICC: 0.574
##   Unadjusted ICC: 0.574
## Summary descriptive statistics
library(e1071)    # for skewness
## Warning: package 'e1071' was built under R version 4.5.2
##
## Attaching package: 'e1071'
## The following object is masked from 'package:ggplot2':
##
##     element
# RMSSD function
rmssd <- function(x) {
  x <- x[!is.na(x)]
  if (length(x) < 2) return(NA_real_)
  sqrt(mean(diff(x)^2))
}

ema_person_stats <- df_emo_exh %>%
  group_by(person) %>%
  summarise(
    n_obs   = sum(!is.na(emotional_exhaustion)),
    mean    = mean(emotional_exhaustion, na.rm = TRUE),
    median  = median(emotional_exhaustion, na.rm = TRUE),
    sd      = sd(emotional_exhaustion, na.rm = TRUE),
    skew    = skewness(emotional_exhaustion, na.rm = TRUE, type = 2),
    rmssd   = rmssd(emotional_exhaustion),
    .groups = "drop"
  )

print(ema_person_stats)
## # A tibble: 18 × 7
##    person n_obs  mean median    sd    skew rmssd
##    <fct>  <int> <dbl>  <dbl> <dbl>   <dbl> <dbl>
##  1 re1      179 23.4    20   13.8   1.17   15.7
##  2 re2      146 25.0    19.5 20.0   1.38   16.2
##  3 re3      191 23.0    20   13.9   0.783  13.9
##  4 re4      178 47.6    50   16.8   0.0770  7.95
##  5 re5      180 70.3    70    9.21 -0.0607 12.0
##  6 re6      170 61.3    63   14.5  -0.599  16.4
##  7 re7      169 52.9    56   21.3  -0.299  25.1
##  8 re8      191 45.9    40   18.9   0.460  23.2
##  9 re9      215  9.89    4   14.1   2.10    8.79
## 10 re10     188 53.0    53   18.1   0.256  12.4
## 11 re11     187 30      29   10.1   0.460  12.7
## 12 re13     192 21.3    21    7.17  1.18    9.34
## 13 re14     189 35.4    29   25.5   0.698  31.8
## 14 re15     183 33.4    29   17.9   0.738  18.0
## 15 re16     136 72.7    71   10.3   0.251  12.8
## 16 re17     158 71.4    78.5 23.8  -0.618  30.5
## 17 re18     167 41.0    39   14.4   0.161  16.8
## 18 re19     158 54.3    54    9.91  0.490  11.8
ema_summary_table <- ema_person_stats %>%
  summarise(
    across(
      c(mean, median, sd, skew, rmssd),
      list(
        Mean = ~ mean(.x, na.rm = TRUE),
        SD   = ~ sd(.x, na.rm = TRUE)
      ),
      .names = "{.col}_{.fn}"
    )
  ) %>%
  pivot_longer(everything(),
               names_to = c("Statistic", "Level"),
               names_sep = "_",
               values_to = "Value") %>%
  pivot_wider(names_from = Level, values_from = Value)

print(ema_summary_table)
## # A tibble: 5 × 3
##   Statistic   Mean     SD
##   <chr>      <dbl>  <dbl>
## 1 mean      42.9   19.0
## 2 median    41.4   21.4
## 3 sd        15.5    5.17
## 4 skew       0.479  0.696
## 5 rmssd     16.4    6.99
bat_vars <- c(
  "BAT_Exhaustion",
  "BAT_Cognitive_impairment",
  "BAT_Emotional_impairment",
  "BAT_Mental_distance"
)

bat_long <- df_model %>%
  select(person, all_of(bat_vars)) %>%
  pivot_longer(
    cols = all_of(bat_vars),
    names_to = "scale",
    values_to = "value"
  )

bat_person_stats <- bat_long %>%
  group_by(scale, person) %>%
  summarise(
    n_obs   = sum(!is.na(value)),
    mean    = mean(value, na.rm = TRUE),
    median  = median(value, na.rm = TRUE),
    sd      = sd(value, na.rm = TRUE),
    skew    = skewness(value, na.rm = TRUE, type = 2),
    rmssd   = rmssd(value),
    .groups = "drop"
  )

print(bat_person_stats)
## # A tibble: 72 × 8
##    scale                    person n_obs  mean median    sd    skew rmssd
##    <chr>                    <chr>  <int> <dbl>  <dbl> <dbl>   <dbl> <dbl>
##  1 BAT_Cognitive_impairment re1        5  6.2     6   1.10   1.29   1.12
##  2 BAT_Cognitive_impairment re10       5 10.8    11   1.79   0.0524 1.87
##  3 BAT_Cognitive_impairment re11       4  6       5.5 2.45   0.544  3.79
##  4 BAT_Cognitive_impairment re13       6  5.5     6   1.76  -0.165  2.10
##  5 BAT_Cognitive_impairment re14       4  8       8   0.816  0      0.816
##  6 BAT_Cognitive_impairment re15       6  8.17    8.5 1.47  -0.418  1.95
##  7 BAT_Cognitive_impairment re16       6  5.5     6   1.38  -1.37   2.05
##  8 BAT_Cognitive_impairment re17       5  3.8     3   1.30   1.71   1.12
##  9 BAT_Cognitive_impairment re18       5  7.6     8   0.548 -0.609  0.707
## 10 BAT_Cognitive_impairment re19       5 10.2    10   0.837 -0.512  1.22
## # ℹ 62 more rows
bat_summary_table <- bat_person_stats %>%
  group_by(scale) %>%
  summarise(
    across(
      c(mean, median, sd, skew, rmssd),
      list(
        Mean = ~ mean(.x, na.rm = TRUE),
        SD   = ~ sd(.x, na.rm = TRUE)
      ),
      .names = "{.col}_{.fn}"
    ),
    .groups = "drop"
  ) %>%
  pivot_longer(
    cols = -scale,
    names_to = c("Statistic", "Level"),
    names_sep = "_",
    values_to = "Value"
  ) %>%
  pivot_wider(names_from = Level, values_from = Value)

print(bat_summary_table)
## # A tibble: 20 × 4
##    scale                    Statistic    Mean    SD
##    <chr>                    <chr>       <dbl> <dbl>
##  1 BAT_Cognitive_impairment mean       7.90   2.33
##  2 BAT_Cognitive_impairment median     7.81   2.39
##  3 BAT_Cognitive_impairment sd         1.27   0.477
##  4 BAT_Cognitive_impairment skew       0.429  1.01
##  5 BAT_Cognitive_impairment rmssd      1.52   0.754
##  6 BAT_Emotional_impairment mean       7.28   2.36
##  7 BAT_Emotional_impairment median     7.19   2.52
##  8 BAT_Emotional_impairment sd         1.43   0.539
##  9 BAT_Emotional_impairment skew       0.0685 1.20
## 10 BAT_Emotional_impairment rmssd      1.66   0.635
## 11 BAT_Exhaustion           mean       8.95   2.37
## 12 BAT_Exhaustion           median     9.06   2.30
## 13 BAT_Exhaustion           sd         1.33   0.637
## 14 BAT_Exhaustion           skew      -0.181  0.795
## 15 BAT_Exhaustion           rmssd      1.64   1.00
## 16 BAT_Mental_distance      mean       7.81   2.15
## 17 BAT_Mental_distance      median     8      2.38
## 18 BAT_Mental_distance      sd         1.66   0.636
## 19 BAT_Mental_distance      skew      -0.116  1.07
## 20 BAT_Mental_distance      rmssd      2.05   0.986
range(df_emo_exh$emotional_exhaustion, na.rm = TRUE)
## [1]   0 100
ema_range <- df_emo_exh %>%
  summarise(
    min = min(emotional_exhaustion, na.rm = TRUE),
    max = max(emotional_exhaustion, na.rm = TRUE),
    range = max - min
  )

print(ema_range)
##   min max range
## 1   0 100   100
bat_ranges <- df_model %>%
  summarise(
    Exhaustion_min = min(BAT_Exhaustion, na.rm = TRUE),
    Exhaustion_max = max(BAT_Exhaustion, na.rm = TRUE),

    Cognitive_min = min(BAT_Cognitive_impairment, na.rm = TRUE),
    Cognitive_max = max(BAT_Cognitive_impairment, na.rm = TRUE),

    Emotional_min = min(BAT_Emotional_impairment, na.rm = TRUE),
    Emotional_max = max(BAT_Emotional_impairment, na.rm = TRUE),

    Distance_min = min(BAT_Mental_distance, na.rm = TRUE),
    Distance_max = max(BAT_Mental_distance, na.rm = TRUE)
  )

print(bat_ranges)
## # A tibble: 1 × 8
##   Exhaustion_min Exhaustion_max Cognitive_min Cognitive_max Emotional_min
##            <dbl>          <dbl>         <dbl>         <dbl>         <dbl>
## 1              3             15             3            15             3
## # ℹ 3 more variables: Emotional_max <dbl>, Distance_min <dbl>,
## #   Distance_max <dbl>
bat_long_ranges <- df_model %>%
  select(
    BAT_Exhaustion,
    BAT_Cognitive_impairment,
    BAT_Emotional_impairment,
    BAT_Mental_distance
  ) %>%
  pivot_longer(everything(), names_to = "scale", values_to = "score") %>%
  summarise(
    min = min(score, na.rm = TRUE),
    max = max(score, na.rm = TRUE),
    range = max - min,
    .by = scale
  )

print(bat_long_ranges)
## # A tibble: 4 × 4
##   scale                      min   max range
##   <chr>                    <dbl> <dbl> <dbl>
## 1 BAT_Exhaustion               3    15    12
## 2 BAT_Cognitive_impairment     3    15    12
## 3 BAT_Emotional_impairment     3    13    10
## 4 BAT_Mental_distance          3    13    10
```
